# Polyploidy is widespread in Microsporidia

**DOI:** 10.1128/spectrum.03669-23

**Published:** 2024-01-12

**Authors:** Amjad Khalaf, Mara K. N. Lawniczak, Mark L. Blaxter, Kamil S. Jaron

**Affiliations:** 1 Tree of Life, Wellcome Sanger Institute, Cambridge, United Kingdom; Broad Institute, Cambridge, Massachusetts, USA

**Keywords:** Microsporidia, polyploidy, tetraploidy, k-mer, k-mer spectra, sequencing data

## Abstract

Microsporidia are obligate intracellular eukaryotic parasites with an extremely broad host range. They have both economic and public health importance. Ploidy in microsporidia is variable, with a few species formally identified as diploid and one as polyploid. Given the increase in the number of studies sequencing microsporidian genomes, it is now possible to assess ploidy levels across all currently explored microsporidian diversity. We estimate ploidy for all microsporidian data sets available on the Sequence Read Archive using k-mer-based analyses, indicating that polyploidy is widespread in Microsporidia and that ploidy change is dynamic in the group. Using genome-wide heterozygosity estimates, we also show that polyploid microsporidian genomes are relatively homozygous, and we discuss the implications of these findings on the timing of polyploidization events and their origin.

**IMPORTANCE:**

Microsporidia are single-celled intracellular parasites, distantly related to fungi, that can infect a broad range of hosts, from humans all the way to protozoans. Exploiting the wealth of microsporidian genomic data available, we use k-mer-based analyses to assess ploidy status across the group. Understanding a genome’s ploidy is crucial in order to assemble it effectively and may also be relevant for better understanding a parasite’s behavior and life cycle. We show that tetraploidy is present in at least six species in Microsporidia and that these polyploidization events are likely to have occurred independently. We discuss why these findings may be paradoxical, given that Microsporidia, like other intracellular parasites, have extremely small, reduced genomes.

## INTRODUCTION

Microsporidia are eukaryotic, obligately intracellular, spore-forming parasites (1), with a broad range of hosts including protozoans, arthropods, and vertebrates (2–5). They were first discovered in 1857 as the cause of a disease known as “pébrine” in silkworms (*Bombyx mori*, Lepidoptera) which had decimated the silk industry from France to China (6–8). In 2001, the first microsporidian genome was published, belonging to *Encephalitozoon cuniculi*, a parasite of the European rabbit (*Oryctolagus cuniculus*) used as a model in infection studies (9). More recently, many studies have targeted microsporidian genomes, and microsporidia have become a valuable model for studying the evolution of parasitism (10–15). Microsporidia have the smallest eukaryotic genomes, having undergone extreme reduction, losing many metabolic pathways, such as glycolysis and lipid biosynthesis (11, 16–19), and lacking mitochondrial genomes because of reductive evolution of mitochondria to form mitosomes (20).

Understanding genome ploidy is critical for correct genome assembly and interpretation, and in Microsporidia, it is also central to understanding their life cycles and parasitism. However, ploidy level has been identified in a few microsporidian species only (9, 21–30). In part, this is due to the fact that microsporidians are thought to undergo cryptomitosis, where the condensation and separation of chromatin into distinct chromosomes are unclear (31, 32). This is further complicated by microsporidia being extremely small and difficult to culture, making isolation of their nuclei from their hosts challenging.

Given that Microsporidia are thought to descend from a sexual fungal lineage (related to the ancestor of Rozellomycota) (33), one possibility is that they retain, in general, ploidy similar to that of their ancestors. In fungi, sexual individuals cycle between different ploidy levels (haploid and diploid, or diploid and tetraploid). Two compatible partners with the lower ploidy level will recognize one another and fuse to form a zygote with the higher ploidy level. When triggered by the right conditions, the zygote undergoes meiosis to produce spores, which may be mono- or di-karyotic (34). Like Basidomycota, some Microsporidia, including *Pleistophora debaisieuxi*, *Astathelohania contejeani*, *Ameson michaelis*, and *Toguebayea baccigeri* (35–39), also cycle between unikaryons and dikaryons (referred to as diplokaryons in Microsporidia), although we do not know if this is associated with ploidy change. Imaging of fixed material has shown evidence for the occurrence of putative events that mirror the fungal sexual cycle in Microsporidia, such as gametogenesis, plasmogamy, karyogamy, and meiosis (36, 40–52).

In contrast, many microsporidian species have been reported to be strictly monokaryotic (e.g., *Encephalitozoon cuniculi*) or strictly diplokaryotic [e.g., *Vairimorpha ceranae* (previously known as *Nosema ceranae*)] for their full life cycle (36, 40, 53, 54). In seven species with monokaryotic spores (*Encephalitozoon cuniculi*, *Nematocida parisii*, *Nematocida* sp1, *Trachipleistophora hominis*, *Hamiltosporidium tvaerminnensis*, *Hamiltosporidium magnivora*, and *Pseudoloma neurophilia*) detected patterns of heterozygosity suggest these species are diploid (9, 21–25, 28, 29). On the other hand, tetraploidy was recently shown to occur in a species with diplokaryotic spores (*Vairimorpha ceranae*, previously known as *Nosema ceranae*) (26). The latter observation was suggested to be associated with the diplokaryon and that the microsporidian diplokaryon may behave like the morphologically similar diplokaryon in diplomonads (26, 55). Perplexingly, both monokaryotic and diplokaryotic spores have been identified for *Spraguea* isolates, with evidence suggesting that monokaryotic spores are diploid, and diplokaryotic spores are monoploid (27, 56, 57). Monoploidy has also been suggested to occur in *Ordospora colligata* (30).

Nevertheless, all current observations relating to ploidy of Microsporidia are restricted to a few species and collected using different methods. Given the wealth of microsporidian-sequencing data now available, it is possible to assess ploidy levels across a wider diversity of taxa. Here, we survey all microsporidian genomic sequencing data sets available publicly in the Sequence Read Archive (SRA) (58) using k-mer-based analyses to make a statement about ploidy across microsporidian phylogeny. Our results suggest that polyploidy is widespread in Microsporidia. By measuring heterozygosity within species at a genome-wide level, we show that the homeologous genomes within polyploids are relatively homozygous. We discuss whether polyploidy is a recent occurrence, and what processes may have given rise to it multiple times.

## MATERIALS AND METHODS

### Download of NCBI SRA data sets

On 7 April 2023, we downloaded all microsporidian whole-genome DNA data sets available in the NCBI SRA (58) using the SRA ToolKit (version 3.0.3, SRA Toolkit Development Team 2023). This retrieved 217 samples from 46 different species (Tables S1 and S2).

### Ploidy estimation

To estimate ploidy, we generated a k-mer spectrum for each sample using Jellyfish (version 2.2.10) with k-mers of length 21 (59). We analyzed these k-mer spectra with Genomescope2 (version 2.0) and Smudgeplot (version 0.2.5) (60) for all the samples. Genomescope2 uses a k-mer spectrum from unassembled read sets to infer genome size and heterozygosity, and can compare the fit of models of various ploidies to the k-mer spectra. Smudgeplot is a visualization technique that receives as input k-mer pairs where each k-mer is paired with those differing by one SNP. The k-mers in each pair are named A and B, with A being the k-mer with coverage greater than or equal to B. In a polyploid genome, k-mers A and B can be found in one or more genomic copies. For example, in a triploid, only the combination AAB can be found, but in a tetraploid, both AAAB and AABB are possible. Ploidy can be inferred by calculating the proportional coverage of B in relation to the total coverage in each k-mer structure combination [see reference (60)]. To ensure that the ploidy estimates were reliable, we only considered data sets where the monoploid (1*n*) coverage was estimated to be over 20-fold, and a good model fit in GenomeScope2 was achieved. For a full explanation of how to interpret GenomeScope2 and Smudgeplot outputs, refer to the Smudgeplot Github page or reference (60).

### Ribosomal small subunit sequence reconstruction

For a representative readset of each of the 16 species surveyed (Table S3), we used PhyloFlash (version 3.3b1, with the -emirge flag) to reconstruct an approximation of its ribosomal small subunit RNA (SSU) sequences (61, 62). We note that this process will collapse homeologous haplotypes and within-species variation. The identity of the reconstructed SSUs was confirmed by using NCBI Blast against NCBI NT (63).

### Phylogeny

We used the reconstructed SSU sequences for the 16 species for which we estimated ploidy, and an additional 116 SSU sequences from related microsporidian species on NCBI NT (Table S4), to generate a phylogeny with *Rozella allomycis* (Rozellomycota) as the outgroup. The sequences were aligned using MAFFT (mafft --maxiterate 1000 --globalpair) (64). The phylogeny was inferred by generating a consensus maximum-likelihood tree using IQ-TREE (version 2.2.2.3) (65), with a GTR+F+I+R6 nucleotide substitution model, 1,000 ultrafast bootstrap replicates, and 1,000 bootstrap replicates for the SH-like approximate likelihood ratio test (iqtree -b 1000 -alrt 1000 -m GTR+F+I+R6). The model was chosen using IQ-TREE’s best-fit model finder according to Bayesian information criterion. The resultant tree was annotated using Toytree and InkScape (version 1.2.2) to show the position of tetraploid and diploid species.

We also used the reconstructed SSU sequences for the 16 species for which we estimated ploidy to generate a smaller phylogeny with *Rozella allomycis* (Rozellomycota) as the outgroup. We aligned the sequences using MAFFT (mafft --maxiterate 1000 --globalpair) (64). The phylogeny was inferred by generating a consensus maximum-likelihood tree using IQ-TREE (version 2.2.2.3) (65), with the GTR+F+I+R6 nucleotide substitution model, 1,000 ultrafast bootstrap replicates, and 1,000 bootstrap replicates for the SH-like approximate likelihood ratio test as above. The alignment of this smaller data set had better fit to a more parameterized model according to IQ-TREE’s best-fit model finder. However, we used the same model used in the larger alignment to maintain consistency. The resultant tree was annotated using Toytree and InkScape (version 1.2.2) to show the position of tetraploid and diploid species.

## RESULTS

### Polyploidy is widespread in Microsporidia

**Fig 1:**
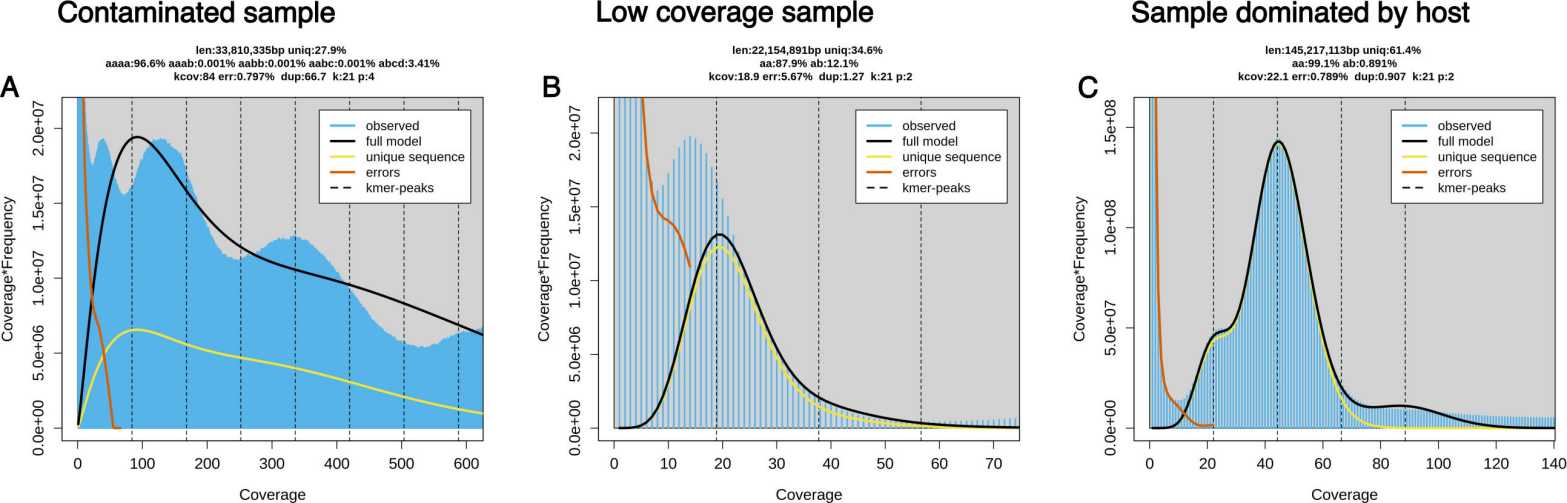
Examples of SRA data sets that were not analyzed further. (**A**) GenomeScope2-transformed linear plot of *Vairimorpha ceranae* (SRR7178080) as an example of a contaminated sample where the pattern coverage peaks are distorted by host contamination, and a good model fit is not achieved. (**B**) GenomeScope2-transformed linear plot of *Encephalitozoon cuniculi* (SRR122315) as an example of a low coverage sample. (**C**) GenomeScope2-transformed linear plot of *Ordospora colligata* (SRR18286429) as an example of a sample that is dominated by host data, showing the predicted genome size to be ~150 Mb.

**Fig 2:**
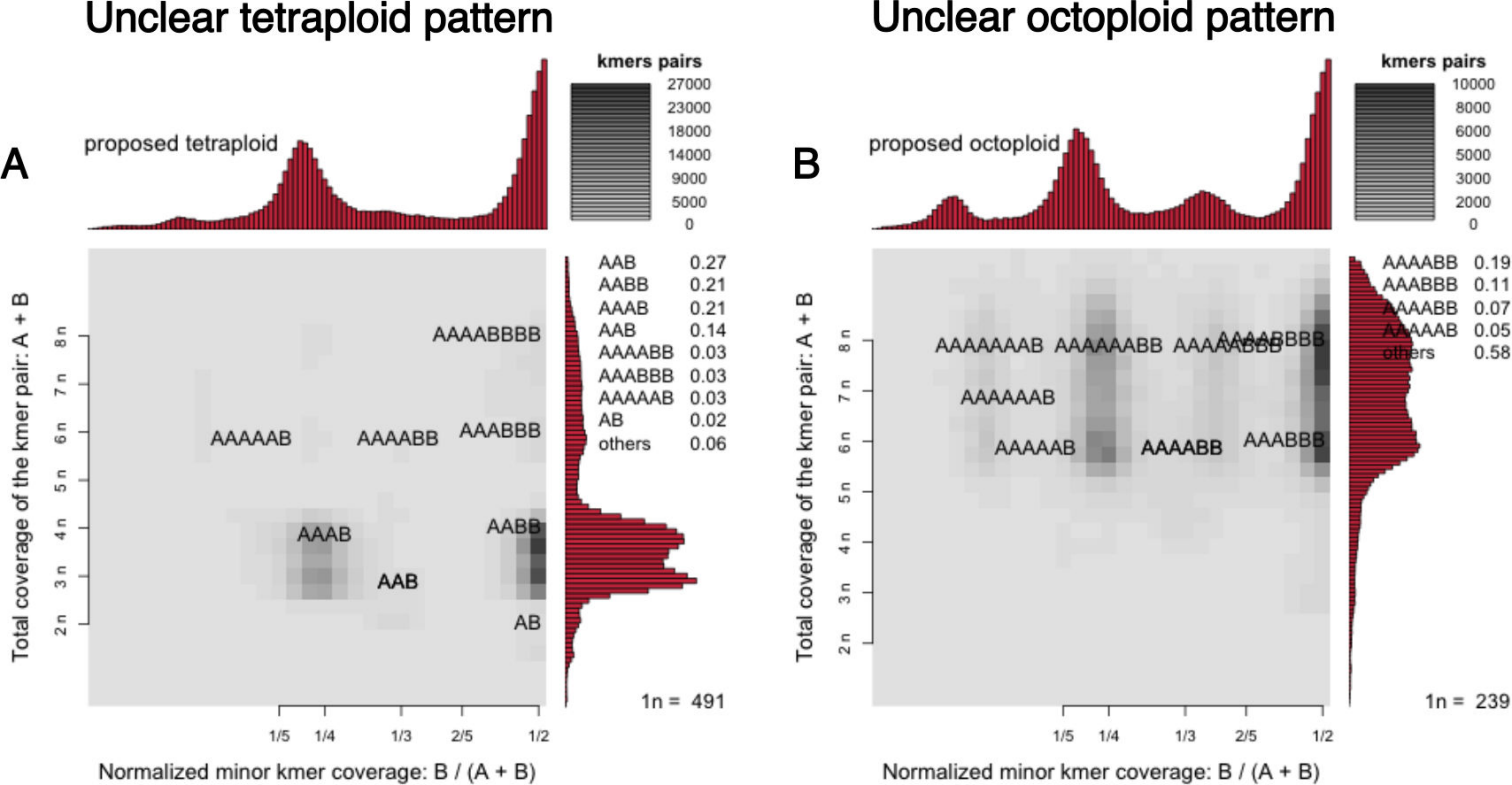
Samples with Smudgeplot patterns that were difficult to interpret. (**A**) *Vairimorpha ceranae* (SRR18590836) Smudgeplot with a smeared tetraploid pattern between 4*n* and 3*n* coverage. (**B**) *Vairimorpha ceranae* (SRR18590837) Smudgeplot with a smeared octoploid pattern between 6*n* and 8*n* coverage. These patterns remain difficult to interpret using current data.

We estimated ploidy across 217 unassembled readsets from 46 species from the NCBI SRA using Genomescope2 and Smudgeplot (60). We filtered out samples with contamination, low coverage samples, and samples that consisted of dominant host peaks (Fig. 1). We also filtered out three samples with unresolved Smudgeplot patterns that we were not able to interpret (Fig. 2). The filtered-out samples and the reasons they were filtered out are in Table S1.

**Fig 3:**
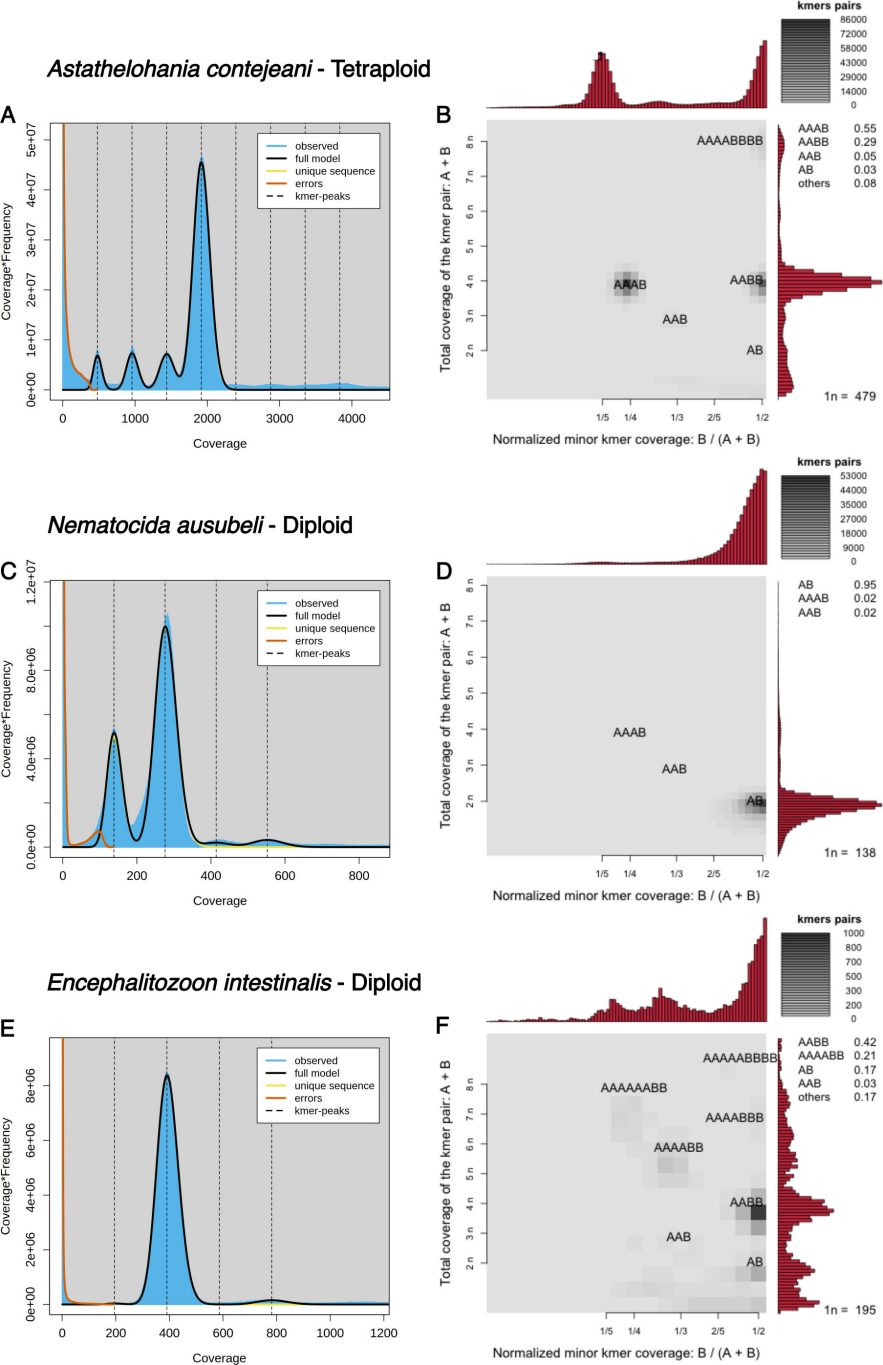
Ploidy estimates for Microsporidia species. (**A and B**) GenomeScope2 and Smudgeplot results for *Astathelohania contejeani* (SRR8476226) estimating tetraploidy. (**C** and **D**) GenomeScope2 and Smudgeplot results for *Nematocida ausubeli* (SRR350188) estimating diploidy. (**E and F**) GenomeScope2 and Smudgeplot results for *Encephalitozoon intestinalis* (SRR24007516) estimating diploidy with exceptionally high homozygosity.

After filtering, we retained 66 samples (from 16 species), all of which showed either diploid or tetraploid Smudgeplot patterns and varying levels of heterozygosity. For example, the GenomeScope2 plot for *Astathelohania contejeani* shows four peaks at 1*n*, 2*n*, 3*n*, and 4*n* coverage (Fig. 3A), while the Smudgeplot shows strong signal at 1/4 and 1/2 normalized minor k-mer coverage (Fig. 3B). These patterns are indicative of tetraploidy. In contrast, the GenomeScope2 plot for *Nematocida ausubeli* shows only two peaks at 1*n* and 2*n* coverage (Fig. 3C), while the Smudgeplot shows strong signal only at 1/2 normalized k-mer coverage (Fig. 3D). This is indicative of diploidy. For the Encephalitozoonidae surveyed in this study, GenomeScope2 plots were interpreted as indicating a diploid genome with extremely reduced heterozygosity (<0.5%). For example, *Encephalitozoon intestinalis* (Fig. 3E) had a single, major k-mer coverage peak (~400-fold in Fig. 3E) and very low signals at half coverage (~200-fold in Fig. 3E) and double coverage (~800-fold in Fig. 3E). The Smudgeplot (Fig. 3F) confirms highly homozygous diploidy, as some signal is seen at 1/2 normalized minor k-mer coverage with ~400-fold (2*n*) coverage. The signal at 1/2 normalized minor k-mer coverage with ~800-fold (4*n*) coverage is likely to derive from imperfect duplications in the genome. This pattern is not consistent with haploidy, triploidy, tetraploidy, or any other higher ploidies. Figures for all 66 samples are available in Table S2. Multiple samples, an average of four per species, were analyzed for many species (Table S2). Where multiple samples were available for a nominal species, they showed a consistent ploidy signal.

**Fig 4:**
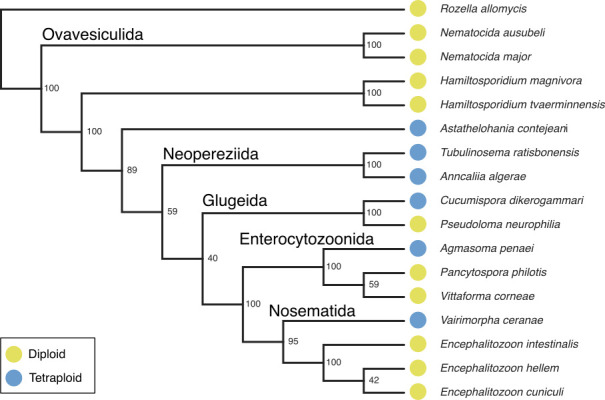
A cladogram of microsporidian species with confident ploidy estimates, rooted on *Rozella allomycis* [diploid, (66)]. The cladogram was inferred from ribosomal small subunit sequences using IQ-TREE (version 2.2.2.3), with a GTR + F + I + R6 nucleotide substitution model.

**Fig 5:**
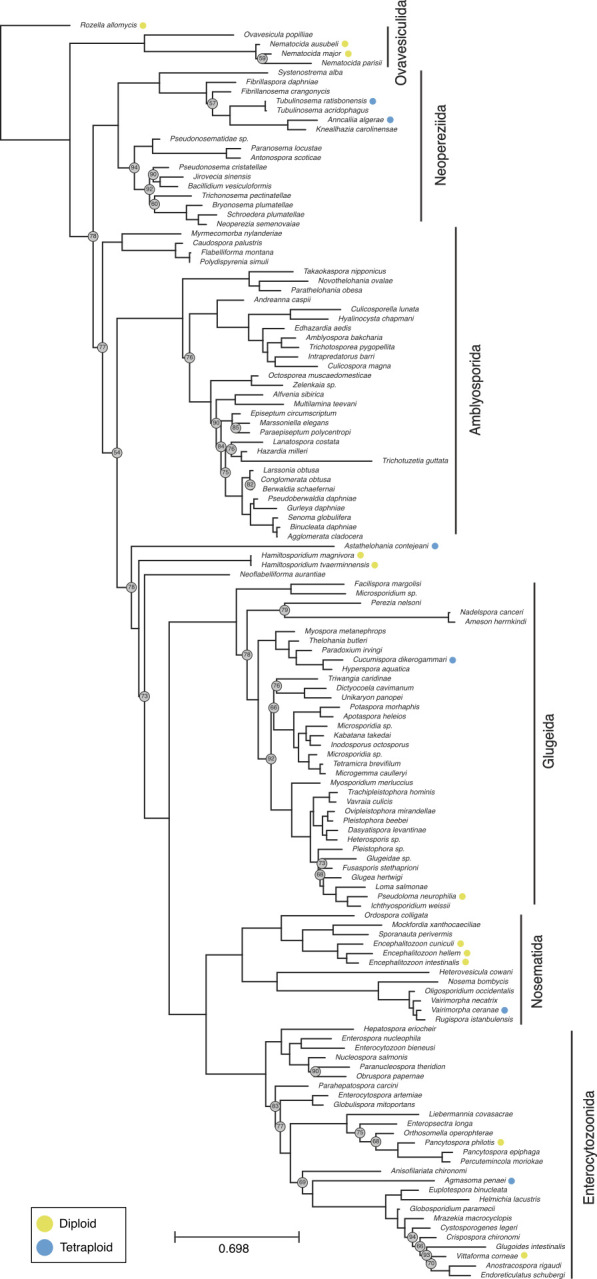
Phylogeny of Microsporidia, rooted on *Rozella allomycis* [diploid, (66)]. The phylogeny was inferred from ribosomal small subunit sequences using IQ-TREE (version 2.2.2.3), with a GTR + F + I + R6 nucleotide substitution model. Nodes with bootstrap values <95% are indicated with gray circles.

Mapping the ploidy estimates to the phylogeny estimated from the SSU locus demonstrated that only diploid species were identified in the clade Ovavesiculida, and no data were available for the clade Amblyosporida. We identified six tetraploid species from three of the six major microsporidian clades (Nosematida, Glugeida, and Neopereziida), and the orphan lineage represented by *Astathelohania contejeani* (12). Neither diploidy nor tetraploidy was monophyletic on the phylogeny. This implies multiple events of gain of polyploidy (five events, if the last common ancestor of Microsporidia is assumed to have been diploid, and only tetraploidy gains are permitted) or gain and loss of polyploidy (e.g., one gain and three losses if the last common ancestor of the clade defined by *Astathelohania* and *Varimorpha* gained tetraploidy) (Fig. 4 and 5).

### Most tetraploid genomes are more than 96% homozygous

**Fig 6:**
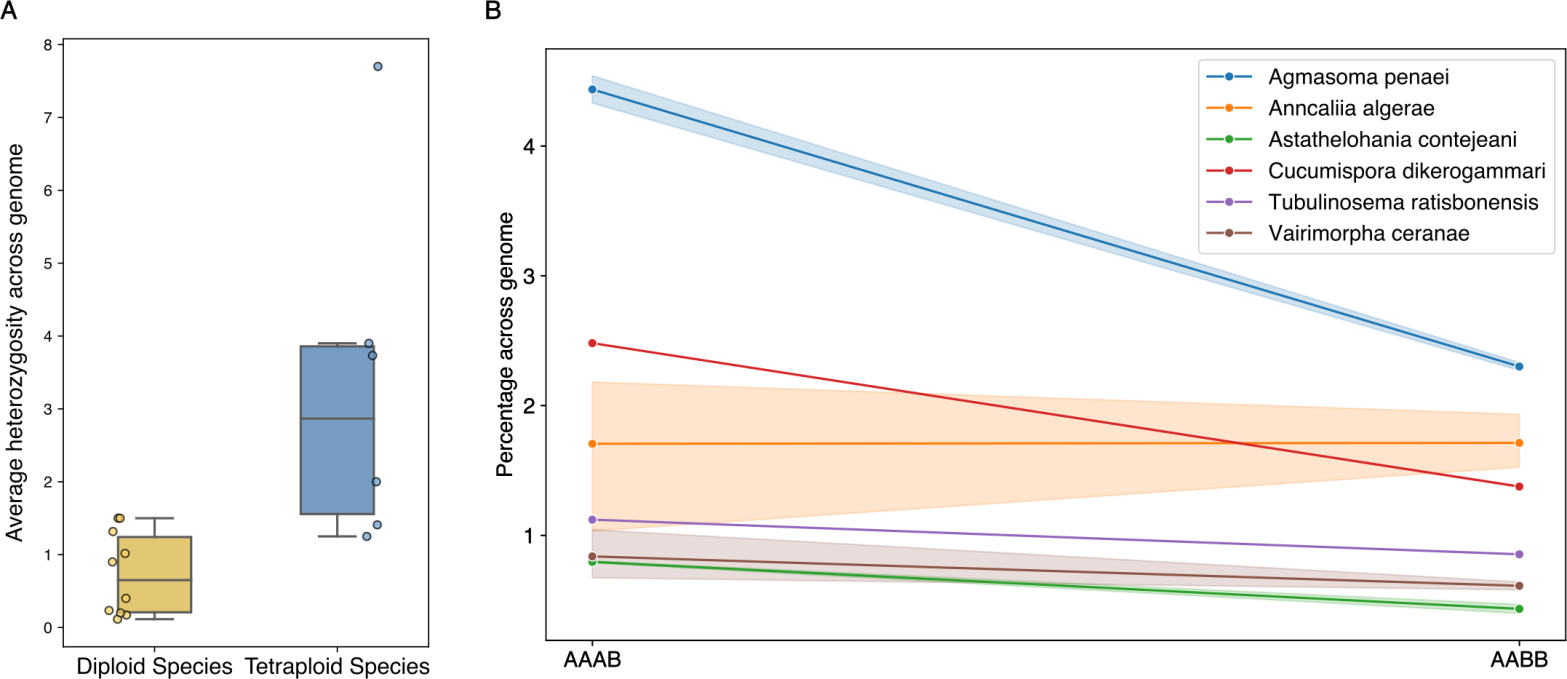
Most diploid and tetraploid genomes are highly homozygous, with the frequency of AAAB k-mers greater than or equal to the frequency of AABB in nearly all cases. (**A**) Boxplot of mean heterozygosity across the genome per species, calculated using GenomeScope2 heterozygosity estimates (1% of AA for diploid species and 1% of AAAA for tetraploid species). (**B**) Line graph showing mean AAAB and AABB heterozygous k-mer patterns across the genome for each of the six tetraploid species. The shaded region represents the value range observed in the independent samples of each species.

We estimated genome-wide heterozygosity across all samples of the 16 species for which we were able to estimate ploidy. Genome-wide heterozygosity in tetraploids varied almost eightfold, ranging from an average of ~8% in *Agmasoma penaei* to an average of ~1% in *Vairimorpha ceranae*. In each sample, we also estimated which k-mer patterns best represent the observed heterozygosity. We found that the frequency of AAAB k-mers (where only one of the four genomic copies carries a different allele) was greater than or equal to the frequency of AABB k-mers (where two of the genomic copies carry an allele, and the other two copies carry another allele) in nearly all cases (Fig. 6).

## DISCUSSION

We have used publicly available genomic data to show that polyploidy is widespread in Microsporidia, with tetraploid species present in three of the six major clades [Nosematida, Glugeida, and Neopereziida (12)], and the orphan lineage represented by *Astahelohania contejeani*.

Many intracellular parasites have reduced genomes compared to free-living relatives across the tree of life (19, 67–69). Genome size reduction is observed in some Microsporidia, and indeed *Encephalitozoon* species have the smallest known assembled eukaryotic genomes, at ~2 Mb (23). However, many Microsporidia have genomes that are of a similar size to those of their sister group the Fungi (modal genome size ~50 Mb; see https://goat.genomehubs.org, search for “Fungi”) (70), and thus genome size reduction among microsporidia is not a universal evolutionary trajectory. Indeed, frequent events of polyploidization, which imply step increases in effective genome span, show that reductive evolution is not dominant. The reduction of the genomes of some intracellular parasites may be driven by evolutionary pressures to reduce replication time and energy consumption, to maintain a small cell size, and through streamlining due to reliance on host-provided metabolism. Small cell size may be particularly important in parasites which exit their host cells through lysis, as the number of spores they can produce will be limited by the relative sizes of their cells and host cells. In-line with this, *Nematocida displodere*, which releases its spores through lysis, has a smaller genome than its counterpart *Nematocida parisii*, whose spores are released through exocytosis (18, 71). However, our sample size is small, and while spores of the tetraploid species are on average larger than those of the diploids, this difference is not significant (Fig. S1).

If polyploidy arises relatively frequently, and the immediate fitness costs of the larger genome and larger spore size are low, polyploid lineages may arise and persist for short periods of evolutionary time before being driven to extinction. Our limited sampling across Microsporidia precludes examination of the relative persistence of polyploids. Additionally, polyploidy may have a positively selected role in rescuing inbred or asexual microsporidians from accumulated lethal mutations and in generating allelic diversity in hybrid lineages. There are no clear differences in haploid genome size, or ecological factors, such as habitats, hosts, and transmission mechanisms that distinguish polyploid species from diploids in our data (Fig. S2 to S5). Additionally, tetraploidy was identified in both species with diplokaryotic spores and species with monokaryotic spores, suggesting that tetraploidy is not associated with the diplokaryon as had been previously indicated (26) (Fig. S6). While the mechanism of the origin of polyploidy also remains uncertain, it is evident that ploidy is dynamic in Microsporidia.

The majority (>96%) of sites in the polyploid genomes are highly homozygous, with the frequency of AAAB k-mers (where one of the four genomic copies differs from the other three) being greater than or equal to the frequency of AABB k-mers (where the two alternate alleles are equally present) in almost all cases. Auto-polyploidy is *a priori* highly plausible, given the intracellular niche of Microsporidia and the presumed rarity of partners (for sex as well as for allo-polyploidization). In an auto-polyploid, we would expect the homeologous chromosomes to show divergence commensurate with the cumulative effect of neutral mutation over time and generations. In the species we assessed as diploid, we identified heterozygosities between 0.5% and 1.5%, which are in accordance with previous heterozygosity estimates for *Encephalitozoon* and *Nematocida* species (21, 22). In the tetraploids, heterozygosity ranged from 1.2% to 4%. This in itself suggests that the homeologous genome copies in the tetraploids are more different from each other than one would expect from very recent auto-polyploidy. Alternatively, if these tetraploids are recent and derived from allo-polyploidy, the parental genomes are likely to have been as or more divergent than the levels we measured between alleles within the diploids. In both models, residual meiotic recombination between homeologous chromosomes and ongoing inter-homeolog gene conversion would tend to reduce observed divergence between homeologs and thus measured heterozygosity. It is difficult to fully interpret the patterns observed without knowledge of microsporidian mutational and recombination rates, the rate of gene conversion between homeologs, the age of the polyploidization events, and the frequency of meiosis in tetraploid species’ life cycles. It is of course possible that different tetraploids were created by different routes.

Progress in disentangling the origins and dynamics of polyploidy in Microsporidia is constrained by current short-read whole-genome data. Phased, chromosomally assembled genome sequences will be critical in distinguishing between different models and also in furthering genomic understanding of microsporidian biology. Ongoing reference-quality genome sequencing and genomic surveillance programs, such as the Darwin Tree of Life project (72) and BIOSCAN, often identify Microsporidia as cobionts when sequencing host species. These initiatives offer an unrivaled opportunity for the generation of high-quality, phased, long-read microsporidian genomes from a variety of hosts and will soon allow us to explore questions like the timing of polyploidization events, their mode, and their genomic impact.

## Data Availability

All data used in this study are publicly available in the NCBI Sequence Read Archive (SRA). Please see Tables S1 and S2 for the list of accession numbers.

## SUPPLEMENTAL MATERIAL

Supplemental material spectrum.03669-23-s0001.pdf Tables S1, S3, and S4; Figures S1 to S6; and Text S1.

Table S2 spectrum.03669-23-s0002.pdf SRA accession numbers, species name, linear GenomeScope2 plots, transformed GenomeScope2 plots, and Smudgeplot plots of the 66 samples for which we were able to estimate ploidy in this study.

ASM does not own the copyrights to Supplemental Material that may be linked to, or accessed through, an article. The authors have granted ASM a non-exclusive, world-wide license to publish the Supplemental Material files. Please contact the corresponding author directly for reuse.
