# Supplementary material for: Polyploidy is widespread in Microsporidia
Source: Microbiol Spectr. 2024 Jan 12;12(2):e03669-23. doi: 10.1128/spectrum.03669-23 (PMC10845963; doi:10.1128/spectrum.03669-23)
Supplement: Supplemental material — Tables S1, S3, and S4; Figures S1 to S6; and Text S1. [file spectrum.03669-23-s0001.pdf]

Polyploidy is widespread in Microsporidia

Amjad Khalaf*, Mara K. N. Lawniczak, Mark L. Blaxter, Kamil S. Jaron

Tree of Life, Wellcome Sanger Institute, Cambridge CB10 1SA, UK

ORCID

Amjad Khalaf	0000-0003-1297-1181
Mara K. N. Lawniczak	0000-0002-3006-2080
Mark L. Blaxter	0000-0003-2861-949X
Kamil S. Jaron	0000-0003-1470-5450

* Corresponding author E-mail: ak37@sanger.ac.uk

This PDF file includes:

- Supplementary Tables 1, 3 and 4
- Supplementary Figure 1 to Supplementary Figure 6
- Supplementary Text 1

Supplied as separate files:

- Supplementary Table 2

Table S1: SRA accession numbers, species name, and reason for exclusion of the SRA samples for which we were not able to reliably estimate ploidy in this study

Accession	Species	Reason for Filtering Out
SRR17317290	Vairimorpha ceranae	Contaminated sample
SRR18590837	Vairimorpha ceranae	Smudgeplot pattern unclear, cannot be interpreted with current data.
SRR24032065	Alternosema sp. JBojko-2023a	No evident peaks
SRR18339014	Anncaliia algerae	Low coverage
SRR18590836	Vairimorpha ceranae	Smudgeplot pattern unclear, cannot be interpreted with current data.
SRR23214359	Enteropsectra breve	Smudgeplot pattern unclear, cannot be interpreted with current data.
SRR16954900	Hamiltosporidium tvaerminnensis	Sample dominated by host
SRR16954907	Hamiltosporidium tvaerminnensis	Contaminated sample
SRR18339013	Anncaliia algerae	No evident peaks
SRR23214362	Nematocida ausubeli	Low coverage
SRR23214354	Nematocida homosporus	Contaminated sample
SRR23214366	Nematocida minor	Contaminated sample
SRR23214357	Nematocida parisii	Contaminated sample
SRR23214356	Nematocida parisii	Contaminated sample
SRR23214352	Nematocida parisii	Contaminated sample
SRR23214351	Nematocida parisii	Contaminated sample
SRR23214361	Nematocida parisii	Contaminated sample
SRR23214360	Nematocida parisii	Contaminated sample
SRR23214365	Nematocida sp. AWRm77	Contaminated sample
SRR23214349	Nematocida sp. AWRm78	Contaminated sample
SRR23214364	Nematocida sp. AWRm79	Contaminated sample
SRR23214348	Nematocida sp. AWRm80	Contaminated sample
SRR23214347	Nematocida sp. LUAm1	Contaminated sample
SRR23214346	Nematocida sp. LUAm2	Contaminated sample
SRR23214345	Nematocida sp. LUAm3	Contaminated sample
SRR23214344	Pancytospora epiphaga	Contaminated sample

ERR3155584	Tubulinosema ratisbonensis	No evident peaks
ERR3155585	Tubulinosema ratisbonensis	No evident peaks
SRR926331	Vairimorpha ceranae	Low coverage
SRR926332	Vairimorpha ceranae	Low coverage
SRR926321	Vairimorpha ceranae	Contaminated sample
SRR3673305	Nosema pieriae	No evident peaks
SRR7178080	Vairimorpha ceranae	Contaminated sample
SRR7178077	Vairimorpha ceranae	Contaminated sample
SRR7178078	Vairimorpha ceranae	Contaminated sample
SRR7178079	Vairimorpha ceranae	Contaminated sample
SRR8481863	Nosema granulosis	No evident peaks
SRR8481864	Nosema granulosis	No evident peaks
SRR8481865	Nosema granulosis	No evident peaks
SRR8481866	Nosema granulosis	No evident peaks
SRR8481867	Nosema granulosis	No evident peaks
SRR8481868	Nosema granulosis	No evident peaks
SRR8481869	Nosema granulosis	No evident peaks
SRR8481870	Nosema granulosis	No evident peaks
SRR8481871	Nosema granulosis	No evident peaks
SRR8481872	Nosema granulosis	No evident peaks
SRR8481873	Nosema granulosis	No evident peaks
SRR8481874	Nosema granulosis	No evident peaks
SRR8481875	Nosema granulosis	No evident peaks
SRR8481876	Nosema granulosis	No evident peaks
SRR8481877	Nosema granulosis	No evident peaks
SRR8481878	Nosema granulosis	No evident peaks
SRR8494486	Dictyocoela roeselum	No evident peaks
SRR8494487	Dictyocoela roeselum	No evident peaks
SRR8494488	Dictyocoela roeselum	No evident peaks
SRR8494489	Dictyocoela roeselum	No evident peaks
SRR8494490	Dictyocoela muelleri	No evident peaks
SRR8494491	Dictyocoela muelleri	No evident peaks
SRR8494492	Dictyocoela muelleri	No evident peaks
SRR8494493	Dictyocoela muelleri	No evident peaks
SRR8536193	Vairimorpha ceranae	No evident peaks

SRR9597067	Hamiltosporidium tvaerminnensis	Low coverage
SRR9597064	Hamiltosporidium magnivora	Low coverage
SRR9597066	Hamiltosporidium magnivora	Low coverage
SRR9597070	Ordospora colligata	Sample dominated by host
SRR9597069	Ordospora colligata	Sample dominated by host
SRR9597068	Ordospora colligata	Sample dominated by host
SRR10339155	Enterospora	No evident peaks
SRR10948363	Vairimorpha ceranae	Contaminated sample
SRR10948362	Vairimorpha ceranae	Contaminated sample
SRR12486971	Anncaliia algerae	Low coverage
SRR530631	Anncaliia algerae PRA109	Low coverage
SRR122343	Anncaliia algerae PRA109	No evident peaks
SRR202953	Anncaliia algerae PRA109	No evident peaks
SRR202954	Anncaliia algerae PRA109	No evident peaks
SRR653432	Anncaliia algerae PRA109	Low coverage
SRR653669	Anncaliia algerae PRA109	Contaminated sample
SRR653670	Anncaliia algerae PRA109	Contaminated sample
SRR653429	Anncaliia algerae PRA109	Low coverage
SRR052647	Anncaliia algerae PRA109	No evident peaks
SRR057726	Anncaliia algerae PRA109	No evident peaks
SRR057727	Anncaliia algerae PRA109	No evident peaks
SRR122312	Encephalitozoon cuniculi	Contaminated sample
SRR122313	Encephalitozoon cuniculi	Contaminated sample
SRR122314	Encephalitozoon cuniculi	Contaminated sample
SRR122315	Encephalitozoon cuniculi	Low coverage
SRR122309	Encephalitozoon cuniculi	Contaminated sample
SRR122311	Encephalitozoon cuniculi	Contaminated sample
SRR064420	Edhazardia aedis	No evident peaks
SRR064202	Edhazardia aedis	No evident peaks
SRR064203	Edhazardia aedis	No evident peaks
SRR064416	Edhazardia aedis	No evident peaks
SRR070507	Edhazardia aedis	No evident peaks
SRR070508	Edhazardia aedis	No evident peaks
SRR070509	Edhazardia aedis	No evident peaks

SRR070510	Edhazardia aedis	No evident peaks
SRR070511	Edhazardia aedis	No evident peaks
SRR070512	Edhazardia aedis	No evident peaks
SRR070514	Edhazardia aedis	No evident peaks
SRR071362	Edhazardia aedis	No evident peaks
SRR071363	Edhazardia aedis	No evident peaks
SRR071364	Edhazardia aedis	No evident peaks
SRR071365	Edhazardia aedis	No evident peaks
SRR052646	Vavraia culicis	No evident peaks
SRR062126	Vavraia culicis	No evident peaks
SRR064422	Vavraia culicis	No evident peaks
SRR122342	Vavraia culicis	No evident peaks
SRR064421	Nematocida ausubeli	No evident peaks
SRR068584	Anncaliia algerae	No evident peaks
SRR068585	Anncaliia algerae	No evident peaks
SRR068586	Anncaliia algerae	No evident peaks
SRR068587	Anncaliia algerae	No evident peaks
SRR058694	Vittaforma corneae	Low coverage
SRR058934	Vittaforma corneae	Low coverage
SRR057728	Vittaforma corneae	Low coverage
SRR065289	Vittaforma corneae	No evident peaks
SRR064417	Vittaforma corneae	Low coverage
SRR064418	Vittaforma corneae	No evident peaks
SRR064419	Vittaforma corneae	No evident peaks
SRR070313	Vittaforma corneae	Low coverage
SRR070314	Vittaforma corneae	No evident peaks
SRR062125	Nematocida ausubeli	Low coverage
SRR636827	Encephalitozoon cuniculi	Low coverage
SRR765863	Nosema apis BRL 01	No evident peaks
SRR765864	Nosema apis BRL 01	No evident peaks
SRR902003	Spraguea lophii	No evident peaks
SRR902029	Spraguea lophii	No evident peaks
SRR1596194	Vairimorpha ceranae	Low coverage
SRR926322	Vairimorpha ceranae	Low coverage
SRR926333	Vairimorpha ceranae	Low coverage

SRR540287	Nematocida ausubeli	Low coverage
SRR501104	Nematocida ausubeli	Low coverage
SRR501105	Nematocida ausubeli	Low coverage
SRR1585328	Nematocida ausubeli	Low coverage
SRR058693	Nematocida ausubeli	Low coverage
SRR543736	Nematocida ausubeli	Sample dominated by host
SRR489793	Nematocida ausubeli	Sample dominated by host
SRR401825	Nematocida ausubeli	Low coverage
SRR065290	Nematocida parisii ERTm3	Low coverage
SRR065291	Nematocida parisii ERTm3	No evident peaks
SRR065292	Nematocida parisii ERTm3	No evident peaks
SRR065288	Nematocida parisii ERTm3	No evident peaks
SRR014461	Vairimorpha ceranae BRL01	Low coverage
SRR040448	Enterocytozoon bieneusi H348	Low coverage
SRR18286426	Ordospora colligata	Sample dominated by host
SRR18286429	Ordospora colligata	Sample dominated by host
SRR18286428	Ordospora colligata	Sample dominated by host
SRR18286427	Ordospora colligata	Sample dominated by host
SRR18286425	Ordospora colligata	Low coverage
SRR18286424	Ordospora colligata	Low coverage
SRR18286423	Ordospora colligata	Low coverage

Table S3: Ribosomal small subunit (SSU) sequences reconstructed from unassembled reads using PhyloFlash (with –emirge enabled).

Accession	Species	Reconstructed SSU sequence
SRR23214363	*Nematocida ausubeli*	AGGTTGATTCTGCCTGACATAGACACTAGTCTCTCAGACT<br>AAGCCATGCAAAACGGGCAAACGGAAGGAAGCGTTGTAC<br>AGCTCAAAAGAACAGTTTCAACCGGCTTGGAGGAAAGGG<br>GCGGACATCCGAGGAAACCTTCGGCCAAGACGACGCACG<br>TCGGGGGGCATCCACGGAGAAGCAGGACGCCAGAGCAG<br>CGCAGGGCGCGGCCAAACGGGAGACTGTCCTATTAGCTA<br>GAAGGTAAGGTCAGGGCTTACCTTGGCGACCATGGGATA<br>CGAGGAATTGGGGTTTGGTTTCGGAGAGGGAGTGTGAGG<br>TGCGGCTCCTAGATCCAAGGATCGCAGCAGGCGCGAAAC<br>TTGCTCACTTCCCGGGGGGAGAAGCAGTGAGGAGACATG<br>CGGGGAAAGAGCGGGCAGCAAAGGAGCGCGCGAGGAGC<br>GATTGGAGGGCAAGACTGGTGCCAGCAGCCGCGGTAATA<br>CCAGCTTCAAAAGTGTCTGTGGTGTTTGTTGTGATTAAAAG<br>GTCCGCAGTCGGGGAGAGAGGCCGTGGGGAAAGCAGGC<br>GGCTCAAAGGGAGAGGCAAGCACGGAAAGAACGCGTCAA<br>GAGGGGCAGGCGGGGGCAGGAGAATTCAGCAGCCAGAG<br>GTGAAATTCGCAGACTTGCTGGGGGCGGACAGAGGCGAA<br>GGCGCCTGCCAAGGACGCTTTCATTGATCAGGGACGAAG<br>GCCGGAGGATCGAAGACGATTAGAGACCGTTGTAGTTCC<br>GGCGGTAAACGGTGCCGACACGGGTGTCTGCGGCGGCG<br>GCGCAGCGGGCGCCCGGAGAGAAATCGAGTGCGAGGGC<br>TTTGGGGAAAGTACAGTCGCAAGACGGAAACTTAAACGAA<br>ATTGACGGAAGGACACCACCAGGAGTGGAGCGTGCGGCT<br>TAATTTGACTCAACACGGGGCACTTTACCGCTGGAAGACG<br>CCAGAGGGATCGGCGCGAGATTTGGCAGAAAGTGGTGCA<br>TGGCCGCTCCTGGCCCGTGGGGTGACCTGTCAGGTTGAA<br>TCCGCTAACGGGCGCGATCCGCGCGCCTTGACTGCGCGG<br>CAGGAAGGCGGACGGCCGGCGCGCAGCCGGAGGAGGG<br>CGGGCGATAGCAGGTCCGTGATGCCCTTTGAAGCAGCGG<br>GCTGCACGCGCGCTACAGTGCGGGCAGGAGGGCCGCGC<br>CGAGAGGCGCGCGCAGAGCGGGCCGCAGGGGATGCCCG<br>CGAGGGAGCGCGGGCTGAACGCGGAATTCCCAGTACCC<br>GCGGGTCACCAGCCCGCGGAGACAGCGTCCCTGTCCTTT<br>GTACACACCGCCCGTCGCTATCTGAGATGGGCGCGCGCG<br>GCAAGCGCGGGGGCGCGCGAGCTGCCGCGCCTAGATTG<br>GATAAAAGTCGTAACAAGGTTTCCGTAGGAGAACCTGCGG<br>AAGGATC
SRR17622377	Nematocida major	AGGTTGATTCTGCCTGACATAGACACTAGTCTCTCAGACT<br>AAGCCATGCAAAACGGGCAAACGGAAGGAAGCGTTGTAC<br>AGCTCAAAAGAACAGTTTCAACCGGCTTGGAGGAAAGGG<br>GCGGACATCCGAGGAAACCTTCGGCCAAGACGACGCACG<br>TCGGGGGGCATCCACGGAGAAGCAGGACGCCAGAGCAG<br>CGCAGGGCGCGGCCAAACGGGAGACTGTCCTATTAGCTA<br>GAAGGTAAGGTCAGGGCTTACCTTGGCGACCATGGGATA<br>CGAGGAATTGGGGTTTGGTTTCGGAGAGGGAGTGTGAGG<br>TGCGGCTCCTAGATCCAAGGATCGCAGCAGGCGCGAAAC

		TTGCTCACTTCCCGGGGGGAGAAGCAGTGAGGAGACATG<br>CGGGGAAAGAGCGGGCAGCAAAGGAGCGCGCGAGGAGC<br>GATTGGAGGGCAAGACTGGTGCCAGCAGCCGCGGTAATA<br>CCAGCTTCAAAAGTGTCTGTGGTGTTTGTTGTGATTAAAAG<br>GTCCGCAGTCGGGGAGAGAGGCCGTGGGGAAAGCAGGC<br>GGCTCAAAGGGAGAGGCAAGCACGGAAAGAACGCGTCAA<br>GAGGGGCAGGCGGGGGCAGGAGAATTCAGCAGCCAGAG<br>GTGAAATTCGCAGACTTGCTGGGGGCGGACAGAGGCGAA<br>GGCGCCTGCCAAGGACGCTTTCATTGATCAGGGACGAAG<br>GCCGGAGGATCGAAGACGATTAGAGACCGTTGTAGTTCC<br>GGCGATAAACGATGCCAACTCGGGGGCGTGCGGCCGGG<br>AGGGCGCGGGCGCCCGGAGAGAAATCGAGTTCGAAGGC<br>TTTAGGGAAAGTACAGTCGCAAGGCAGAAATTTAAACGGA<br>AATTGGCGGATTAATACACCAGGAGTGGAGCATGCGGCTT<br>AATTTGACTCAACACGGGGCAGCTTACCGCTGGAAGACG<br>CTGGGGGGATCGGGGCGAGATTTGGCAGAGAAGTGGTG<br>CATGGCCGCTCTTGGTCCGTGGGGTGACCTGTCAGGTTG<br>AATCCGCTAACGGGCGCGACGCGCGTGCGCCAAAAGAAA<br>GAGGGTGCAGACGGCCGGCGCGCAGCCGGAGGAGGGC<br>GGGCGATAGCAGGTCCGTGATGCCCTTTGAAGCAGCGGG<br>CTGCACGCGTGTTACAGTGTGCGCAGGAGGGGTGCGCCG<br>AGAGGCGCTGCAGCAAACGCGGGCGGGGGATGCCGGCG<br>AGGGAGCGCGGGCTGAACACGGAATTTCCAGTACCCGCG<br>GGTCACCAGCCCGCGGAGACAGCGTCCCTGTTTTTTGTAC<br>ACACCGCCCGTCGCTATCTGAGATGGGCGGCGCGGCAAG<br>CGCGGACACGCGCGAGCTGCTGCGCCTAGATTGGATAAA<br>AGTCGTAACAAGGTTTCCGTAGGAGAACCTGCGGAAGGAT<br>CACTTC
SRR16954902	Hamiltosporidium magnivora	CACCAGGTTGATTCTGCCTGACGAGGATGCTTGTCTCTGG<br>GATTAAGCCATGCAAGTCTGGTGAAGCGAAAGTGGAACTG<br>CGAACGGCTCAGTAGAACGGTGATTATTTAATCGTGTAGG<br>AAGGATAACCGCGGGAAACTGTGGCTAATAACACGAGTAA<br>GACGCCGACCCATCAGTTTTATCGTACGGTAAGGGCGTAC<br>GATGGCTTTAACGGGTACGGGGGATCAGGGTTTGATTCC<br>GGAGAGGGAGCCTGAGAAACGGCTACCAGGTCTAAGGAC<br>AGCAGCAGGCGCGAAACTTGCCCAATTGGAGAGAGGCAG<br>TTATGAGACGTATATTTTGTAACCGGGGTAGAATACCGGT<br>GATTGACTGGAGGGCAAGTCTGGTGCCAGCAGCCGCGGT<br>AATTCCAGCTCCAGGAGTGTCATAGTGCATTGCTGCATTT<br>AAAAGGTCCGTAGTCAGTGTTGACAGGGATGCTAGAAGAC<br>ACTTTCTTCATGGAGTGTATTGGCATTCTAGGTTAGCATGT<br>AGGAGCGGAAGAGGGCGACTGTATTGCGTAGCGAGAGGT<br>GAAAGTTGACGACCTACGTAGGACAAACCGAAGTGAAAGC<br>TGTCGTCCAGTACGTTTCCGGTGATCAAGGACGTAAGCCG<br>GAGGAGCAAAGGTGATTAGAGACCCCTGTAGTTCCGGCC<br>GTAAACTATGCCAACTGGGTGTTACGTAGTGGTACCCACG<br>AGAAATCAAGTATATGGGCTATGGGGATAGTACGATCGCA<br>AGATTGAAACTTGAAGAAATTGACGGAAGGACACCACCAG<br>GAGTGGAGTGTGCGGCTTAATTTGACTCAACGCGGGACA<br>ACTTACCAGGGCCAGTTGTATGACGAATCTATGATGAGTA<br>CGACTAAGTGGTGCATGGCCGTTCACAACATGTGAGGTGA<br>CTTTTAGGTTTATTGCTGTAATGTGTGAGACCCTCAGCTAC<br>GGCGACTGGGGACTATAAGTTTCAGGAAGGAGGGGGCTA<br>TAACAGGTCTGTGATGCCCTTAGATGTTCTGGGCTGCACG<br>CGCACTACAATGTTATTTCTAGGTATATTGTAAAGATAAAG<br>AATAACTCGGTTGGGATTGCGTTCTGTAATGGACGCATGA<br>ACCAGGAATTCCTAGTAGTCGCGTGTCACTAACGGGCGAC<br>GACTGCGTCCCTGTTCTTTGTACACACCGCCCGTCGTTAT

		CTAAGATGGTGCTATGTTCGAAGAGGGTTTTCCTTCTGAG<br>GACGTAGGATTAGATTGGATACAAGTCGTAACAAGGTTGC<br>TGTAGGAGAACCTGCAGCAGGATCAGTGATAGTACTTTTA<br>TTGTGTAGTTTTTTCATATGAAACTCATACAAGGGATCACT<br>TGGCTCCGTATCCGAGGAAGGCCGGAATAGTTTCCGAGA<br>ACTAAGGCCGAACGGCATTTGTAAATGCCTGACTACCCCC<br>TGGACTTAAGCATATTATTAAGGGGAGGAACAAAAACTAA<br>CTAGGATTTCTGTAGTAGCGGCGAGCGAACAAGAAATAGC<br>CCCGAATGTAAGCCTCCGGGCATTGTTAAGATCGTCGTAA<br>CGAATTACCGGGACAGGTAAGCCATAGAGGGTGATAGCC<br>CCGTAGTGACGAGTAATGGGCAGAGTAGGGTTGCTTGGT<br>AATGCAGCCTGAAGAGGTGGTGGGTTCCATCTAAGGCTAA<br>ATATGAGCGGAGACCGATAGCGTAATAGTACAGCGATGGA<br>AAGGTGAAAAGCTAAAAAGTGCAAAGACGTGAAATTGTAT<br>TGAGTATCCTGATAACAGGACCCGTCTGAAACACGGACCA
SRR16954899	Hamiltosporidium tvaerminnensis	AGGTTGATTCTGCCTGACGAGGATGCTTGTCTCTGGGATT<br>AAGCCATGCAAGTCTGGTGAAGCGAAAGTGGAACTGCGA<br>ACGGCTCAGTAGAACGGTGATTATTTAATCGTGTAGGAAG<br>GATAACCGCGGGAAACTGTGGCTAATAACACGAGTAAGAC<br>GCCGACCCATCAGTTTTATCGTACGGTAAGGGCGTACGAT<br>GGCTTTAACGGGTACGGGGGATCAGGGTTTGATTCCGGA<br>GAGGGAGCCTGAGAAACGGCTACCAGGTCTAAGGACAGC<br>AGCAGGCGCGAAACTTGCCCAATTGGAGAGAGGCAGTTA<br>TGAGACGTATATTTTGTAACCGGGGTAGAATACCGGTGAT<br>TGACTGGAGGGCAAGTCTGGTGCCAGCAGCCGCGGTAAT<br>TCCAGCTCCAGGAGTGTCATAGTGCATTGCTGCATTTAAA<br>AGGTCCGTAGTCAGTGTTGACAGGGATGCTAGAAGACACT<br>TTCTTCATGGAGTGTATTGGCATTCTAGGTTAGCATGTAGG<br>AGCGGAAGAGGGCGACTGTATTGCGTAGCGAGAGGTGAA<br>AGTTGACGACCTACGTAGGACAAACCGAAGCGAAAGCTGT<br>CGTCCAGTACGTTTCCGGTGATCAAGGACGTAAGCCGGA<br>GGAGCAAAGGTGATTAGAGACCCCTGTAGTTCCGGCCGT<br>AAACTATGCCAACTGGGTGTTACGTAGTGGTACCCACGAG<br>AAATCAAGTATATGGGCTATGGGGATAGTACGATCGCAAG<br>ATTGAAACTTGAAGAAATTGACGGAAGGACACCACCAGGA<br>GTGGAGTGTGCGGCTTAATTTGACTCAACGCGGGACAACT<br>TACCAGGGCCAGTTGTATGACGAATCTTTGATGAGTACGA<br>CTAAGTGGTGCATGGCCGTTCACAACATGTGAGGTGACTT<br>TTAGGTTTATTGCTGTAATGTGTGAGACCCTCAGCTACGG<br>CGACTGGGGACTATAAGTTTCAGGAAGGAGGGGGCTATA<br>ACAGGTCTGTGATGCCCTTAGATGTTCTGGGCTGCACGCG<br>CACTACAATGTTATTTCTAGGTATATTGTAAAGATAAAGGA<br>TAACTCGGTTGGGATTGCGTTCTGTAATGGACGCATGAAC<br>CAGGAATTCCTAGTAGTCGCGTGTCACTAACGGGCGACG<br>ACTGCGTCCCTGTTCTTTGTACACACCGCCCGTCGTTATC<br>TAAGATGGTGCTATGTTCGAAGAGGGTTTTCCTTCTGAGG<br>ACGTAGGATTAGATTGGATACAAGTCGTAACAAGGTTGCT<br>GTAGGAGAACCTGCAGCAGGATCA
SRR8476225	Astathelohania contejeani	GGTTGATTCTGCCTGACGTGGAAGCTATTCTTTAAGATTAA<br>GCCATGCATGTGTAGAATGAAGTGAAGCCATTAGGTGGAA<br>CAGCGAAAAGCTCAGTAATACAATCATTATTTGGTCTAC<br>AAGATATAGAATAACCTTGATAAATTAAGGCTAAAGCTATT<br>GTAGAATAAGAGATTGACCTATCAGCTAGTATGTAGGGTA<br>AGGGCCTACGTAGGCGATGACGGGTAACGGGGAATTAGG<br>GTTCTATTCCGGAGAAGGAGGCTGAGAGATGGCTACTAG<br>GTCTAAGGAGAGCAGCAGGCGCGAAACTTACCCAATGCT<br>ATTTAGTAGTGTAGAGTGTAGTGAATATTCTAATGGAGAGG

		CTAGTAAAGCAACCATTGTAATTCCAGCAAATCTATTACGA<br>GTATTGCTGCAATTAAAAAGTTCGTAGTTGATTATTGTAAT<br>AATCTTGTGATAAGTATTAAATTATGATTAATGAAAGCCAT<br>GGAAGGAAATAGGATTAAATAGGGAGGGGTGAAATCTGTA<br>GATCTATTTAGGACTAACTGAAGCGAAAGCGATTTTCTATG<br>TGGTATTTGACAATCAAGAACGAAAGCCGGAGTATCGAAG<br>ACGATTAGAGACCGTCGTAGTTCCGCGCAGTAAACTATGT<br>TATATTATTTGTAGTATATATGAGTATATATTATGGATATAT<br>AGAAATTAAGATATTATGAACTTTGGGGATAGTACGAACGC<br>AAGTTTTAAACTTAAATGAAATTGACGGAAGGACACACCAG<br>GAGTGGAGTGTGCGGTTTAATTTGACTCAACGCGGGACAA<br>CTTACCATTTTTAGAAGTGAATATGAATGATATTTATCATGA<br>TTTTACTATGAGTGGTGCATGGCCGTTAACAATACGTGAT<br>GTGAATTTTGAATTTGAATGAGTTATATATGAAAGTATATAA<br>TTCTGTATTAGTGTTAAATCCACCAATGTGTGAGACCCATA<br>TACTAATTTTGAGTTAGTAGACAGATGATGTAAATCATAGG<br>AAGGGATGGGCGATAACAGGTCAGTGATGCCCTTAAATGA<br>AATGGGCGACACGCGCACTACAATAGAATGATATATTATT<br>CTAAGGAGTTGGGATTATTAATATGTAAATTTAATATGAAC<br>AAGGAATTCCTAGTAATATTTTTTTTGTGATGTTAACAACGA<br>GATATGAATATGTCCCTGTCCTTTGTACACACCGCCCGTC<br>GTTATCTCAGATGGATATTAGGGTGAAATATTAATATAATA<br>GAGAACTCTAATAACTAAATAAGGTACAAGTCGTAACAAG<br>GTTGACCTAAGTGAACTTGGGGCAGGATCA
ERR3154977	Tubulinosema ratisbonensis	GATTGATTCTGTCTGTTATATGTGCTAGTGTCGAAGATTTA<br>GCCATGCATGCTTTACGAATTCACAAGAAGAAGTGGCGGA<br>CAGCTCAGTAATACAGTTATAATATACTCTCTACTAAAGGA<br>TAACCGCGGTAAGCTGCGGGTAAAACTTCAAGCTATGTGT<br>TTCATGAAAGAATTTATAGTTCAGAGTGTTAGTTTCTGGCC<br>TATTAGTTAGTAGGTAATGTAAGGGATTACCTAGACTATTA<br>TGGGTAACGGGAGATGAATGTCTGATACCGGAGAGGAAG<br>CCTTAGAAACCGCTTTCACGTCCAAGGATGGCAGCAGGC<br>GCGAAACTTACCCAATCGTTTTTTAAGACGGAGGTAGTTAT<br>GACACATGTGAATTATTCACGAGGAAGATCAATAAATCAGA<br>TTTTTTTTACTGGAGGGCAAGTCTGGTGCCAGCAGCCGCG<br>GTAATTCCAGCTCCAGTAGTGCATATACATGCTGTAGTTAG<br>AAAGTTTGTAGCCGTATAAGCTTGAATCAGAGGAAAGGAG<br>GACTCTTAAGGTAAACTTTAACTCTGGTCAATGCTTATATT<br>CGAAGCGGATGGAGGCACTTGTATTCAATAGCGAGAGGT<br>AAAATTTGATGACCTATTGAGGACAATCAGTAGCGAAAGC<br>GAGTGTCTAGTACGCGTTTGAGGGTCAAGAACGTTAGCCG<br>GAGGATCGAAGATGATTAGATACCGTTGTAGTTCCGGCCG<br>TAAACCATGCCTACTTGCCTTGTACTTAGTATGAAGCATAG<br>AGAAATTAAGAGTTTTTGGGCTCTAGGGATAGTAATCCGG<br>CAACGGACAAACTTAAAGAAATTGGCGGAAGGACACCACA<br>AGGAGTGGATTGTGCGGCTTAACTTGACTCAACACGGGAA<br>ATCTTACCAGGGTCTGTATATGTGAGACTGTCCCATTTATG<br>GTGGTACACGATAATATACTGAGTGGTGCATGGCCGTTTT<br>CAACACGTGGGGTGACCTGTCAGGTTTATTCCGGTAACGT<br>GTGAGGTCACAGTATTAATAAGTATTTTAATAGACAGCTAA<br>TGGTAAATTAGAGGAAGTGTGACGATAACAGGTCAGTGAT<br>GCCCTTAGATACCCTGGGCTGCACGCGCAATACATTGAGT<br>AAGTCATTTACAACAGTAATTTTGAAACTTACTCGCTACTG<br>GGATCATCCTTTGTAATTGGGATGTGAACGTGGAATTCCT<br>AGTAATCGTAGCTCACTAAGTTACGATGAATGTGTCCCTGT<br>TCTTTGCACACACCGCCCGTCGCTATCTAAGATGGATGTT<br>GATATGAAATTGCTGCTTGAATGTGAGGTACTTGAGTATTA

		ACAACTAGATAAGATATAAGTCGTAACAAGGCTGCTATAGA<br>AGAATCTGTGGCAGGATCA
SRR489790	Annaliia algerae	CACCAGATTGATTCTGTCTGGTATGTGTGCTAGCGTCAAA<br>GATTTAGCCATGCATGCTTTTCGAACCCTCGTGGGGAGAG<br>GCGGATAGCTCAGTAATACAGTTATAACATAAGCTGCGTG<br>TGTGGATAACCTTGTTAAGATAAGGCTAAGACTTAATAAGT<br>CGCACTTTTGTGAAGAAACGCGACTTGTGCAGCATTGGTT<br>TCTGACCTATCAGTTAGTATGTTCTGTAAGGGAGAACATAG<br>ACTATGACGGGTAACGGGGGATGCACGTCTGATACCGGA<br>GAGGAAGCCTTAGAGACAGCTTTCACGTCCAAGGATGGC<br>AGCAGGCGCGAAACTTACCCAATTGTTTTTTGTGACAGAG<br>GTAGTTATGACGTATTCTTTAGAGAGAGACCTTGTTAGACA<br>TGGTCCATAGCGACTGGAGGGCAAGTCTGGTGCCAGCAG<br>CCGCGGTAATTCCAGCTCCAGTAGTGCATATACATGCTGT<br>AGTTAGAAAGTTTGTAGCCTATTTATGGATTGTTTTAGACA<br>AAAGGACGACTCAAATTGACCTTTCATTTGACTAATGCATG<br>AATGTAGAAGCGATTGAAGGCGATTGTATTCACCAGCCAG<br>AGGTAAAATTTGATGACCTGGTGAGGACACACGGAGGCG<br>AAAGCGATTGCCTAGAGCGTATTCAGTGGTCAAGAACGTA<br>AGCCGGAGGATCAAAGATGATTAGATACCGTTGTAGTTCC<br>GGCCGTAAATTATGCCAACTTGTGCTTCTGCTTCTGCGGA<br>GGCGCATAGAGAAATCAAGAGTTTATGGGCTCTAGGGATA<br>GTAATCCGGCAACGGACAAACTTAAAGAAATTGGCGGAAG<br>GACACCACAAGGAGTGGATTATGCGGCTTAATTTGACTCA<br>ACGCGGGACAACTCACCAGAGCCTATGTGCAGGAGACAG<br>TGAGCTTTGAGAGCGGACTGGATAGTACTTTGAGTGGTGC<br>ATGGCCGTTTGCAACACGTGAGGTGACTTGTCAGGTTTAC<br>TCCGGTAACGTGTGATGTGCTGTATGCAAGTATTTTTGTGA<br>GACTGCAGGCGGTAAGCCTGATGAAGCGGCGCTATAACA<br>GGTCAGTGATGCCCTTGGATGTTCTGGGCTGCACGCGTA<br>ATACAGTGGGAGCTGTAGATATGTATAGGTGGAAAAGTTC<br>CCGAGACTGGGATCATGCTTTGTAAGAAGGATGTGAACGT<br>GGAATTCCTAGTAATCGCTGCTCACTAAGTAGCGATGAAT<br>GAGTCCCTGTTCTTTGCACACACCGCCCGTCGCTATCTGA<br>GATGGATGTTTTTATGAAGATGCTGCTGTTAGAGGCATTTG<br>AGTAAGGACGACTAGATTAGATATAAGTCGTAACAAGGCA<br>GCGGTAGAAGAATCTGCCGCTAGATCATAAT
SRR8495097	Cucumispora dikerogammari	CAGGTTGATTCTGCCTGACGTGGACGCTAGTCTCATAGAT<br>TTAGCCATGCATGTGTAAGCGAACAAGAGGAAGCTGCGG<br>ACTGCTCAGTAACAGACATATAATTTAATCTTTACAGAAAC<br>GAGCGGAATAAACTCAGGAAACAGAGTGCAATACGTAAAA<br>GACGAATTTTTTATTATAAGAAATACGTTTTTAGCTTGAACA<br>AAGCGGTAAAGAATAAGTTGTCAGCCTATCAGTTAGTAAG<br>TAGGGTAAGGGCCTATTTAGACGAAGACGGGTACGGGGA<br>ATTAGAGTTTGATTCCGGAGAGGGAGCCTGAGAAATAGCT<br>ACCAGGTCCAAGGACGGCAGCAGGCGCGAAAATTACCGA<br>AGCTCGAATAGAGGCGGTAGTAATGAGACGTATTAATATA<br>AAACAAGGGTAAAAAACTTGTTAGTAACTGGAGGTCAAGT<br>CTGGTGCCAGCATCCGCGGTAATACCAGCTCCAGGGGTG<br>TCTATGATGATTGCTGCGATTAAAAGGTCCGTAGTCGAATT<br>TATATAATTGTTTGTAATATGCTAGATAAAATAACAGAAAGA<br>ACAATTACTTTAAATGAAAGGAATAGTAAGGGGCTGATTAA<br>TTGAGCAACGAGAGGTGAAATTTGATGACTTGCTTAGGAG<br>AAACAGAGGCGAAAGCGTCAGTCAAGTATAAATCCTATGA<br>TCAAGGACGTAGGCTAGAGTATCGAACACGATTAGATACC<br>GTAGTAGTTCTAGCAGTAAACTATGCCTACACTATCGAATA<br>AAAGTTTGGTAGAAGAGAAATCTTAAGTAGGGCTTTGGGG

		AGAGTACACGCGCAAGCGATAAATTTAAAGGAAATTGACG<br>GAAGAACACCACAAGGAGTGGAGTGTGCGGCTTAATTTGA<br>CTCAACGCGGGACAGCTTACCATACCCGAGGACTATAAGA<br>GTGAATACACGATAAGTCTAAAAGTGGTGCATGGCCGTTA<br>TCGACGAGTGAAGTGATTTTATGGTTAAATCCGACAAGTT<br>GTGAGACCCTTATTTAAATACAGGTATTGTTAAAATACAGG<br>AAGGAAAGGACAAGAACAGGTCAGTGATGCCCTTAGATG<br>GTATGGGCTGCACGCGCACTACAATGGTTATAATAATAAA<br>GATAATTAAAGTATAAATATAATCAAGAGGAATTGAGAACT<br>GAAAAGTTCCCATGAACGAGGAATTGCTAGTAATCGTAGG<br>CTCAGTAAGATACGATGAATATGTCCCTGTTCTTTGTACAC<br>ACCGCCCGTCGTTATCGAAGATGGAGTTTTACCCGAACAA<br>GCTTAAGCGAGTGAGTGTATGATTCTAGATCTGATACAAG<br>TCGTAACAAGGCAGCCGTAGGAGAACCTGCTGCTGGATC<br>AACT
SRR926312	Pseudoloma neurophilia	CAGGTTGATTCTGCCTGACGTGGATGCTAGTTTCATAGAT<br>TAAGCCATGCATGTGTAAGCGAAGCGTAAGTGGAGCGGC<br>GTACGGCTCAGTAACGGGCTACTATTTGATCTCCCTGGGC<br>GGATATCCTCTGTAAACGGAGGGCAAAACGCAAGACAAG<br>CAGCAATTTTGTTGGCTGTCTAACGAAAGTGGGGGAGAGT<br>AAGGAGCCAGCCCATCAGCTAGTAAGTAGGGTAAGGGCC<br>TACTTAGGCAAATACGGGTACGGGGAATTATCGTTTGATT<br>CCGGAGAGGGAGCCTGAGAGATGGCTACCGGGTCCAAG<br>GACAACAGCAGGCGCGAAAATTACCGGAGCCTGAAGTCA<br>GGGCGGTAGTAAGGAGACGTGAGAACAATGTGGGGGTAA<br>AAAACGCACTGGAAACAGGAGGAAAAGACTGGTGCCAGC<br>ACCCGCGGTAATACCAGCTCCTGGAGTGTCTATGGTGATT<br>GCTGCAGTTAAAGCGTTCGTAGTCGGAGCGGAAAGAATG<br>GCGTGACAGACAGCTGCTCAAAGGGTGGTGTGCGCCGTG<br>ATCCCGCGGAATGAGGAGAGTTTTGGGACCAGGCTATTAA<br>ACGGCAAGCGGTGAAATGTGGTGACCCGTTTAGGAGCAA<br>CAGAGGCGAAAGCGCTGGTCAGGGGCTATTCCGATGATA<br>AAGGACGTAGGCTAGAGGATCGAAGACGATTAGAGACCG<br>TTGTAGTTCTAGCAGTAAACGATGCCGATGTTGTGGTGCA<br>GAGTGCAACGCAAGAGAGAAATCTAGTAGGGCCCTGGGG<br>AGAGTACGCGCGCAAGCGAGAAATTTAAAGGAAATTGACG<br>GATGAACACCTCAAATAGTGGAGTGTGCGGCTTAATTTGA<br>CTCAACGCGGGACATCTTACCGGGCCCGACGACCGGACG<br>AGCGTGACACGCGATAGGTCGGAGAGTGGTGCATGGCCG<br>TTAACGACGAGTGGAGTGATCTTTGGGTTAAGTCCGTAAA<br>TTAGTGAGACCCCAGCGGAAGGACAGGTGCGCAACGCAC<br>AGGAAGGATGGGTCAAGGACAGGTCAGTGATGCCCTTAG<br>ATGGCCCGGGCTGCACGCGCACTACAGTGGTCGCCGAAA<br>TTTGGAGAGAGAGGGAAAGGCGATCGAGAGGGAATGAGC<br>TTTGGAAGAGGCTCAGGAACGCGGAATTGTTAGTAATCGC<br>GGGCTCACTAAGACGCGATGAATGCGACCCTGTTCATTGT<br>ACACACCGCCCGTCGTTATCGAAGACGATGCTAGGCGCG<br>AGCAAGGTTTATTTGGCTGAGCGAGCGCAGGGTATTGAAT<br>CTGATGTAAGTCGTAACAAGGTAGCTGTAGGAGAACCTGT<br>AGCTGGATCA
SRR926341	Agmasoma penaei	CACCAGGTTGATTCTGCCTGACGTAGAGGCTAGCCTCAG<br>GGACTAAGCCATGCATTGTTAGTGAAGTTTTTAATGAAACG<br>ACGGACGGCTCAGTAATACTACTTTTAACTAACCTTTTGTA<br>CTAATAATTAAGGGAAACTGTAATTAAAAATCATGAGGATG<br>TGAGGTAGACCTATTAGCTAGTTGGTTGTGTAAAGGACTA<br>CCAAGGCTATAATGGGTAACGGAGATTTAGTGATCGAAAC<br>CGGAGATGGAAGCTGAGAAACGGTTCCAATGTCCAAGGA

		TAGCAGCAGGCGCGAAAATTGCACACTCTTTAATGGGGAT<br>GCAGTTATGAGGTATGACAGAAAGGGTTATCAATAAATAA<br>GATGACGTAAAGCTATTAGAGGGAAAGTTTGGTGCCAGCA<br>GCCGCGGTAATACCAACTCTAAGAGTCTCTATGCGAGTTG<br>CTGCAGTTAAAAAGTCCGTAGTCTTTACGTAATTAAAAATG<br>AATGATCAAGTTTCATATTTTTACGTTTATATATGAGACGGA<br>TTGGGAGCATAGTATAACTGGGTTAAGAATGAAATCTCACT<br>ACCCTAGTTGGACTATCAGAAGCGAAAGCGATGCTCTAAT<br>ACGTACTTTTAGATAAAGGACGAAGGCTAGAGTAGCGAAA<br>GGGATTAGATACCCCTGTAGTTCTAGCAGTAAACTATGCC<br>GACAGAATGTTAGATATATTTCTAGTGTTCAAGGGAAACCT<br>TAAGTGATCGGGCTCTGGGGAGAGTATGCTCGCAAGTGT<br>GAAAATTAAACGAAATTGACGGAGTTACACCACAAGGAGT<br>GGATTGTGCGGCTTAATTTGACTCAACGCGAGGAATTTTA<br>CCAGGGCTGAATATATTTGAGATTGATTACATGAAATATAT<br>TTGAGTGGTGCATGGTCGTTGTAAACTCATGGATTGATCTT<br>AAGTTCAACTGCTAAAATGGGTGAGACTTTCATAAACAGCT<br>ATCTAACAGGTAGAGGAAGGGGAAGGCGATAACAGATCC<br>GTGATGCCCTCAGATGTCCTGGGCTGCACGCGCAATACAT<br>TATGTATATTTCTTATAAATAGATACTACATATTGGGGAATT<br>GACTTTTGTAAATAAGTCATGAACTTGGAATTCCTAGTAAT<br>AATGATTCATCAAGTCATTGTGAATGTGTCCCTGTAGCTTG<br>TACACACCGCCCGTCACTGTCTCAGATGGTTGATGAGATG<br>AAGAGCTTCGGTTCTGAATCTTAACAACTAAATAAGACATA<br>AGTCGTAACAAGGTATCG
SRR23214350	Pancytospora philotis	CGGCGACCGAACGGCGAACGGCTCAGTAATACTGCGACG<br>ATCTGCCCCGCGCGGCCGATAACCGCGGGAAACTGCGGC<br>TAAGAGCGCGGGTTGAGACGCAGCCCTATCAGCTCGTTG<br>GCGGTGTAACGGACCCCCAAGGCGGCGACGGGTGACGG<br>GGGGTCGGGGCCCGACTCCGGAGAGGGAGCCTGAGAGA<br>TGGCTCCCACGTCCAAGGACGGCAGCAGGCGCGGAAATT<br>GCCCACTCCCGCGCGGGGAGGCAGTTACGAGACGTGGC<br>GGAAGGGCGCCCCGCAACGCCGGGCGCGAAGGCGATTG<br>GAGGGCAAGCTCGGTGCCAGCAGCCGCGGTAATACCGAC<br>TCCAAGAGTGTCTATGGTGGATGCTGCAGTTAAAACGTCC<br>GTAGTCGGCACGGCAACGAAGACGCGCGGCTCGAACGC<br>GCGCGCGTTGCCGGACGCGGCGGAGCGGGCAGGGGGC<br>GCGGTATGCGCGGGCGAGAGATGAAACGCCAGGACCCC<br>GCGCGGACCGACGGCGGCGAAGGCGGCGCCCCGGGAC<br>GCGTCTGCGGATCAAGGACGAAGGCCGGAGGATCGAAAG<br>TGATTAGAGACCGCTGTAGTTCCGGCAGTAAACGATGCCG<br>ACAGCGCGGCGCGCGTGCGCCGCGCGGGGGAAACCTTA<br>GTGTGCGGGCTCTGGGGATAGTATGCTCGCAAGCGTGAA<br>AATTAAACGAAATTGACGGAGCTACACCACAAGGAGTGGA<br>TTGTGCGGCTTAATTTGACTCAACGCGGGGCAGCTTACCA<br>GGGCCGCGCGCGCCCGAGACCGCGCGCGCGAGGCGCG<br>CAGGAGTGGTGCATGGTCGTTGCAAGCCGATGGCGCGAG<br>CTTAGGCTTAAGTGCCGGAATGGGCGAGATCCGCGCGAC<br>GGGTGCGCGCCGCACAGGCGGGAGCGGGCGATAACAGA<br>TCAGTGATGCCCTCAGATGCCCTGGGCCGCACGCGCAAT<br>ACACTCCCCGCGCGCGGCGCAGACGGGTCGCGGGGGGC<br>GGGACCGGCGCCTGCAAGGGCGCCGCGAACGAGGAATT<br>CCTAGTAACGGCGGCTCACCAGGCCGCGGTGAATGTGTC<br>CCTGTAGCTTGTACACACCGCCCGTCACTATCTCAGACGG<br>CCGCCGCGGCGAAGGGAGCG

SRR065293	Vittaforma corneae	ACCAGGTTGATTCTGCCTGACGTAGATGCTAGTCTCTAAG<br>ATTAAGCCATGCATGTTTCCGCAATCAGGGACGAATAGCT<br>CAGTAAAACTGCGATGATTTAGTCTGGCTGTGTAGATAACT<br>ACGTGAAAATGTAGCTAAGGGAAGGCAGAATAAGACGCA<br>GGACTATCAGTTAGTTGGTAGTGTAATGGACTACCAAGAC<br>AGTGACGGTTGACGGGAAATTAGGGTTTTGTACCGGAGA<br>GGGAGCCTGAGAGATTGCTCCCACGTCCAAGGACGGCAG<br>CAGGCGCGAAAATTGCCCACTCTTTGCAGGAGGCAGTTAT<br>GAGACGTGAAGATGAGTATCTTGTAAAGAGGGATAGGAGA<br>ATTGGAGGGCAAGTTTGGTGCCAGCAGCCGCGGTAATAC<br>CGACTCCAAGAGTGTGTATGAGAGATGCTGCAGTTAAAAA<br>GTCCGTAGTCATAGAAGGGCAAAGAGAGATGCGAGGTCT<br>CACAGTGCGATGATGGAGGAGCCGATGGGGAACATAGTA<br>TACCAGGGCGAGAGATGAAATGCCAAGACCCCTGGTGGA<br>CTGAGCGAGGCGAAGGCGATGTTCTTGTAGGCATTCGGT<br>GATCAAGGACGAAGGCTGGAGTATCGAAAGTGATTAGATA<br>CCGCAGTAGTTCCAGCAGTAAAAGATGCCGACATGCTCAT<br>TGGACACAGTGGGCAGGGAGAAATCTTAAGAGTTCGGGC<br>TCTGGGGATAGTATGCTCGCAAGGGTGAAAATTAAAGAAA<br>TTGACGGAGCTACACCACAAGGAGTGGATTGTGCGGCTTA<br>ATTTGACTCAACGCGAGGAAACTTACCAGGGCCAAGTATT<br>GTGTAGAAACGAGCAATACAGGAGTGGTGCATGGTCGTT<br>GGAAATTGATGGGATGACTTTGACCTTAAATGGTTGAATG<br>AGTGAGATCTTTTGGACATGTTCCGCACGGAACAGGAAGG<br>AAAAGGCTATAACAGATCCGAGATGCCCTCAGATGCCCTG<br>GGCTGCACGCGCAATACAATAGCAGGTAGAGAGAGAGAC<br>AGGAAGGTGCTCAGATGGACTATGTGCTGTAAGGCACATA<br>CGAAAGAGGAATTCCTAGTAAGTGTGTATCAACAATGGAT<br>ATTGAATAAGTCCCTGTAGCTTGTACACACCGCCCGTCAC<br>TATCTCAGATGTTTTTCAGGATGAAGAGTCCAGGCTCTGA<br>ATAATGAAAAGTAGATAAGATGTAAGTCGTAACAAGGTTGC<br>GGTCGGTGAACCAGCCGCAGGATCATTG
SRR17317295	Vairimorpha ceranae	CACCAGGTTGATTCTGCCTGACGTAGACGCTATTCCCTAA<br>GATTAACCCATGCATGTTTTTGACATTTGAAAAATGGACTG<br>CTCAGTAATACTCACTTTATTTTATGTAAATTTTTAATTAACT<br>ACGTTAAAGTGTAGATAAGATGTTTACAGTAAGAGTGAGA<br>CCTATCAGCTAGTTGTTAAGGTAATGGCTTAACAAGGCTG<br>TGACGGGTAACGGTATTACTTTGTAATATTCCGGAGAAGG<br>AGCCTGAGAGACGGCTACTAAGTCTAAGGATTGCAGCAG<br>GGGCGAAACTTGACCTATGGATTTTATCTGAGGCAGTTAT<br>GGGAAGTAATATTATATTGTTTCATATTTTAAAAGTATATGA<br>GGTGATTAATTGGAGGGCAAATCAAGTGCCAGCAGCCGC<br>GGTAATACTTGTTCCAAGAGTGTGTATGATGATTGATGCA<br>GTTAAAAAGTCCGTAGTTTATTTTTAAGAAGCAATATGAGG<br>TGTACTGTATAGTTGGGAGAAAGATGAAATGTGACGACCC<br>TGACTGGACGAACAGAAGCGAAAGCTGTACACTTGTATGT<br>ATTTTTTGAACAAGGACGTAAGCTGGAGGAGCGAAGATGA<br>TTAGATACCATTGTAGTTCCAGCAGTAAACTATGCCGACG<br>ATGTGATATGTATTAATTTGTATTACATAATAGAAATTTGAG<br>TTTTTTGGCTCTGGGGATAGTATGATCGCAAGATTGAAAAT<br>TAAAGAAATTGACGGAAGAATACCACAAGGAGTGGATTGT<br>GCGGCTTAATTT<br>GACTCAACGCGAGGTAACTTACCAATATTTTATTATTTTGA<br>GAGAACGGTTTTTTGTTTGAGAATGATAATAGTGGTGCATG<br>GCCGTTTTCAATGGATGCTGTGATTAATTTCAACAAGACGT<br>GAGACCCTTATTTTTTATTAAAGACAGACACAATCAGTGTA<br>GGAAGGAAAGGATTAAAACAGGTCCGTTATGCCCTCTGAC<br>ATTTTGGGCTGCACGCGCAATACAATAGATATATAATCTTT

		ATGGGATAATATTTTGTAAGAGATATTTGAACTTGGAATTG<br>CTAGTAAATTTTATTAAATAAGTAGAATTGAATGTGTCCCT<br>GTTCTTTGTACACACCGCCCGTCGCTATCTAAGATGATATA<br>TGTTGTGAAATTAGTGAAAACTACTTAAACAATATGTATTA<br>GATCTGATATAAGTCGTAACATGGTTGCTGTTGGAGAACC<br>ATTAGCAGGATCATAATGATTTTTAAAATTATTTATTTCATA<br>TTATTATTTTTATTTTGCCCACACATGGGATCAATAGGATA<br>CCATAACGATGAAGGTCGTAATAGAATACGAAAGTATTTTA<br>ATATTTACCGAATTAATATTTAATAATATTGATTACCCTT
SRR24007515	Encephalitozoon intestinalis	CATCAGGTTGATTCTGCCTGACGTGGATGCTATTCTCTGG<br>GACTAAGCCATGCATGTTGATGAACCTTGTGGGGGATTGA<br>CGGACGGCTCAGTGATAGTACGATGATTTGGTTGGCGGG<br>AGAGCTGTAACTGCGGGAAACTGCAGGTAGGGGGCTAGG<br>AGTGTTTTTGACACGAGCCAAGTAAGTTGTAGGCCTATCA<br>GCTGGTAGTTAGGGTAATGGCCTAACTAGGCGGAGACGG<br>GAGACGGGGGATCGGGGTTTGATTCCGGAGAGGGAGCCT<br>GAGAGATGGCTACTACGTCCAAGGATGGCAGCAGGCGCG<br>AAACTTGCCTAATCCTTTGGGGAGGCGGTTATGAGAAGTG<br>AGTTTTTTTCGAGTGTAAAGGAGTCGAGATTGATTGGAGG<br>GCAAGTCGGGTGCCAGCAGCCGCGGTAATACCTGCTCCA<br>ATAGTGTCTATGGTGAATGCTGCAGTTAAAAAGTCCGTAG<br>TCTTTTGTATGTCTTTGTTTGGGGGATTATGTCCTGATGTG<br>GATGTAAGAGGTTTGGCAGAGGACGAGGGGCACCGGATA<br>GTTGGGCGAGGGGTGAAATACGAAGACCCTGACTGGACG<br>GACAGAAGCGAAGGCTGTGCTCTTGGACTTATGTGACGAT<br>GAAGGACGAAGGCTAGAGGATCGAAATCGATTAGATACC<br>GTTTTAGTTCTAGCAGTAAACGATGCCGACTGGACGGGAC<br>TATATAGTGTTGTGCATGAGAAATCTTGAGTATGTGGGTTC<br>TGGGGATAGTATGCTCGCAAGAGTGAAACTTGAAGAGATT<br>GACGGAAGGACACCACAAGGAGTGGAGTGTGCGGCTTAA<br>TTTGACTCAACGCGGGGCAACTTACCGGTTCTGAAGCGG<br>GCAGGAGAACGAGGACGGGATGCGCGCGGCGGTGGTGC<br>ATGGCCGTTTGAAATGGATGGCGTGAGCTTTGGATTAAGT<br>TGCGTAAGATGTGAGACCCTTTGACAGTGCTCTTTGGGGC<br>AAGGGAGGAATGGAACAGAACAGGTCCGTTATGCCCTGA<br>GATGAAGCGGGCGGCACGCGCACTACGATAGATGGCGAG<br>GGAGCCTGCTGTGAGGGATGAAGCTGTGTAATGGGCTTC<br>TGAACGTGGAATTCCTAGTAATAACGATTGAACAAGTTGTT<br>TTGAATGGGTCCCTGTCCTTTGTACACACCGCCCGTCGCT<br>ATCTAAGATGACGCAGTGGACGAAGATTGGAAGGTCTGAG<br>TCCTTCGTGTTAGATAAGATATAAGTCGTAACATGGCTGCT<br>GTTGGAGAACCAGCAGCAGGATCAGTATTTG
SRR23560257	Encephalitozoon hellem	CATCAGGTTGATTCTGCCTGACGTGGATGCTATTCTCTGG<br>GGCTAAGCCATGCATGTTTATGAAGCCTTTATGGGGGATT<br>GACGGACGGCTCAGTGATAGTACGATGATTTGATTGGGAG<br>CCTGGATGTAACTGTGGGAAACTGCAGGTAAGTTCTGGG<br>GGTGGTAGTTTGTAGCTACTGCGTACCGAGTAAGTTGTAG<br>GCCTATCAGCTGGTAGTTAGGGTAATGGCCTAACTAGGCG<br>G<br>AGACGGGAGACGGGGGATCAGGGTTTGATTCCGGAGAGG<br>GAGCCTGAGAGATGGCTACTACGTCCAAGGATGGCAGCA<br>GGCGCGAAACTTGCCTAATCCTTATTGGGGAGGCGGTTAT<br>GAGAAGTAAGATGTTTAGCAAGTATAAATTTGTTGTGATTT<br>ACTGGAGGGCAAGTCGGGTGCCAGCAGCCGCGGTAATAC<br>CTGCTCCAGTAGTGTCTATGGTGAATGCTGCAGTTAAAAT<br>GT<br>CCGTAGTTGTTTGTATGTCTTTTGAGTGATGTTTATGGTTT

		TTAGTGGATGTAGTTTTATTGTAGCAGAGGACGAGGGGCA<br>CTGGATAGTTGGGCGAGGGGTGAAATACGAAGACCCTGA<br>CTGGACGAAGAGAAGCGAAGGCTGTGTTCTTGGACTTTTG<br>TGGTGATGAAGGACGAAGGCTAGAGGATCGAAATCGATTA<br>GATACCGTTTTAGTTCTAGCAGTAAACGATGCCGACTGGA<br>CGGGACTGTTTTAGTGTTGTCCGAGAGAAATCTTAAGTAT<br>GTGGGTTCTGGGGATAGTATGCTCGCAAGAGTGAAACTTG<br>AAGAGATTGACGGAAGGACACCACAAGGAGTGGAGTGTG<br>C<br>GGCTTAATTTGACTCAACGCGGGGCAACTTACCGGTTCTG<br>AAGTGAGTGTGAGAGTGTTTTTACATGATGCTTACGGCGG<br>TGGTGCATGGCCGTTTTAAATGGATGGCGTGAGCTTTGGA<br>TTAAGTTACGTAAGATGTGAGACCCTTTTTGACTGTGCTCT<br>ATGGGGCAAGGGAGGAATGGAACAGAACAGGTCCGTTAT<br>GCCCTGAGATGAAGCGGGCGGCACGCGCACTACGATAGA<br>TGCCTATGTGGGCTACTGTGAGGGATGAAGCTGTGTAATG<br>GGCTTCTGAACGTGGAATTCCTAGTAAGAATGATTGAACA<br>AGTTATTTTGAATGTGTCCCTGTCCTTTGTACACACCGCCC<br>GTCGCTATCTAAGATGACGCAGTGGACGAAGATTGAGAG<br>GTCTGAGTCTTTCGTGTTAGATAAGATATAAGTCGTAACAT<br>GGCTGCTGTTGGAGAACCAGCAGCAGGATCAGTA
SRR17858635	Encephalitozoon cuniculi	CCAGGTTGATTCTGCCTGACGTGGATGCTATTCTCTGGGG<br>CTAAGCCATGCATGCTTGTGAACTCTTTGTGGGGGATTAG<br>CGGACGGCTCAGTGATAGCACGATGATTTGTTTGCGGGAT<br>GAGCAGTAGCTGCGGGAAACTGCAGATAGTGGTCTGCCC<br>CTGTGGGGGTTGGCAAGTAAGTTGTGGGCCTATCAGCTG<br>GTAGTTAGGGTAATGGCCTAACTAGGCGCAGACGGGATA<br>CGGGGGATCAGGGTTTGGTTCCGGAGAGGGAGCCTGAGA<br>GATGGCTACTACGTCCAAGGATGGCAGCAGGCGCGAAAC<br>TTGCCTAATCCTTTGGGGAGGCGGTTATGAGAAGTGATGT<br>GTGTGCGAGTGCAAAGGGGTCGCATGTGATTGGAGGGCA<br>AGTCGGGTGCCAGCAGCCGCGGTAATACCTGCTCCAATA<br>GTGTCTATGGTGGATGCTGCAGTTAAAATGTCCGTAGTCT<br>GTTGTGTATGTCTTTGTGTGTGATGTTTGTGGTTGTGTGTG<br>GATGTAGTGATGTGTGTGGCAGAGGACGAGGGGCACTGG<br>ATAGTTGGGCGAGAGGTGAAATGCGAAGACCCTGACTGG<br>ACGAGCGGAAGCGAAGGCTGTGCTCTTGGACTAATGTTG<br>CGATGAAGGACGAAGGCTAGAGGATCGAAATCGATTAGAT<br>ACCGTTTTAGTTCTAGCAGTAAACGATGCCGACTGGACGG<br>GACTGTGTGTGTTGTCCATGAGAAATCTTGAGTATGCGGG<br>TTCTGGGGATAGTATGCTCGCAAGAGTGAAACTTGAAGAG<br>ATTGACGGAAGGACACCACAAGGAGTGGAGTGTGCGGCT<br>TAATTTGACTCAACGCGGGGCAACTTACCGGCTCTGAAGG<br>ATGCCTGTGAGTGCATGGCATGAGGCATGCGGCGGTGGT<br>GCATGGCCGTTTTAAATGGATGGCGTGAGCTTTGTCTTAA<br>GTTGCGTAAGATGTGAGACCCTTTGACGGTGTTCTACGGA<br>GCAAGGAGGGGATGGAAGAGAACAGGTCCGTTATGCCCT<br>GAGATGAGGCGGGCTGCACGCGCACTACGATAGATGGCG<br>CTTCTGCCTGCTGTGAGGGATGAAGCTGTGTAAGGGGCTT<br>CTGAACGTGGAATTCCTAGTAATAGCGGCTGACGAAGCTG<br>CTTTGAATGTGTCCCTGTCCTTTGTACACACCGCCCGTCG<br>CTATCTAAGATGACGCACTGGACGAAGATCGGAAGGTCTG<br>AGTCCTGAGTGTTAGATAAGATATAAGTCGTAACATGGCT<br>GCTGTTGGAGAACCAGCAGCAGGATCAGTAT

Table S4: NCBI NT accession numbers of additional contextual species included in phylogenetic tree.

Accession	Species name
JQ062988	*Ichthyosporidium weissii*
HM626203	*Loma salmonae*
GQ203287	*Glugea hertwigi*
AJ252958	*Pleistophora sp.*
MT006314	*Glugeidae sp.*
MW077214	*Fusasporis stethaprioni*
AF356223	*Ovipleistophora mirandellae*
KX099692	*Pleistophora beebei*
GU183263	*Dasyatispora levantinae*
KC137548	*Heterosporis sp.*
AJ002605	*Trachipleistophora hominis*
XR552272	*Vavraia culicis*
AY530532	*Myosporidium merluccius*
KX364284	*Hyperspora aquatica*
KU163282	*Paradoxium irvingi*
DQ417114	*Thelohania butleri*

HM140491	*Myospora metanephrops*
AY958070	*Nadelspora canceri*
MN935433	*Ameson herrnkindi*
KX856426	*Perezia nelsoni*
HM800849	*Facilispora margolisi*
MF429927	*Microsporidium sp.*
AF356222	*Kabatana takedai*
MH911629	*Inodosporus octosporus*
MF974572	*Microsporidia sp.*
AF364303	*Tetramicra brevifilum*
AY033054	*Microgemma caulleryi*
GQ868443	*Microsporidia sp.*
EU534408	*Potaspora morhaphis*
MG708238	*Apotaspora heleios*
AJ438959	*Dictyocoela cavimanum*
MW377751	*Unikaryon panopei*
JQ268567	*Triwangia caridinae*
GQ206147	*Neoflabelliforma aurantiae*
DQ675604	*Euplotespora binucleata*
GU130406	*Helmichia lacustris*

MN595900	*Globosporidium paramecii*
FJ914315	*Mrazekia macrocyclopis*
AY233131	*Cystosporogenes legeri*
JX915758	*Anostracospora rigaudi*
L39109	*Endoreticulatus schubergi*
GU130407	*Crispospora chironomi*
AF394525	*Glugoides intestinalis*
GU126383	*Anisofilariata chironomi*
FJ389667	*Paranucleospora theridion*
HG005137	*Obruspora papernae*
U78176	*Nucleospora salmonis*
JX101917	*Enterospora nucleophila*
L07123	*Enterocytozoon bieneusi*
HE584635	*Hepatospora eriocheir*
JX915760	*Enterocytospora artemiae*
KT762153	*Globulispora mitoportans*
KX757849	*Parahepatospora carcini*
KX424959	*Pancytospora epiphaga*
LC136798	*Percutemincola moriokae*
EU709818	*Liebermannia covasacrae*

KX360142	*Enteropsectra longa*
AJ302316	*Orthosomella operophterae*
FJ865223	*Mockfordia xanthocaeciliae*
KC172651	*Sporanauta perivermis*
AF394529	*Ordospora colligata*
DQ996241	*Vairimorpha necatrix*
KR704648	*Rugispora istanbulensis*
AF495379	*Oligosporidium occidentalis*
MT510137	*Nosema bombycis*
EU275200	*Heterovesicula cowani*
EU075347	*Binucleata daphniae*
KT950767	*Agglomerata cladocera*
DQ641245	*Senoma globulifera*
MK053815	*Pseudoberwaldia daphniae*
AF439320	*Gurleya daphniae*
AF394527	*Larssonia obtusa*
MH645035	*Conglomerata obtusa*
AY090042	*Berwaldia schaefernai*
AY090067	*Hazardia milleri*
AY326268	*Trichotuzetia guttata*

KX832080	*Lanatospora costata*
AY090041	*Marssoniella elegans*
AY880951	*Paraepiseptum polycentropi*
AY880953	*Episeptum circumscriptum*
KT950766	*Alfvenia sibirica*
KC990122	*Multilamina teevani*
FN794114	*Octosporea muscaedomesticae*
EF537880	*Zelenkaia sp.*
JF826402	*Amblyospora bakcharia*
HM594269	*Trichotosporea pygopellita*
AF027684	*Edhazardia aedis*
AY013359	*Intrapredatorus barri*
AY326269	*Culicospora magna*
AF027683	*Culicosporella lunata*
AF483837	*Hyalinocysta chapmani*
EU664450	*Andreanna caspii*
JF826419	*Novothelohania ovalae*
AY090065	*Parathelohania obesa*
KF110990	*Takaokaspora nipponicus*
AJ252962	*Flabelliforma montana*

AY090069	*Polydispyrenia simuli*
AF132544	*Caudospora palustris*
KR704917	*Myrmecomorba nylanderiae*
EF564602	*Ovavesicula popilliae*
XR001214623	*Nematocida parisii*
GU173849	*Kneallhazia carolinensae*
AF024658	*Tubulinosema acridophagus*
MF278272	*Fibrillaspora daphniae*
AY364089	*Fibrillanosema crangonycis*
AY953292	*Systenostrema alba*
AY135024	*Schroedera plumatellae*
MN512229	*Neoperezia semenovaiae*
AF484691	*Bryonosema plumatellae*
AF484695	*Trichonosema pectinatellae*
MN752317	*Jirovecia sinensis*
AJ581995	*Bacillidium vesiculoformis*
AF484694	*Pseudonosema cristatellae*
JX463178	*Pseudonosematidae sp.*
AY305324	*Paranosema locustae*
AF024655	*Antonospora scoticae*

NG017174	*Rozella allomycis*

Text S1: Newick file format of generated phylogenetic tree.

(SRR17317295_Vairimorpha_ceranae:0.0216381998,(((((((((SRR23560257_Encephalitozoon_hellem:0.1270261617,SRR24007515_Encephalitozoon_intestinalis:0.0579490963)100:0.0456705591,SRR17858635_Encephalitozoon_cuniculi:0.1222391079)100:0.1689587732,(FJ865223_Mockfordia_xanthocaeciliae:0.2351313696,KC172651_Sporanauta_perivermis:0.1712250092)100:0.1447538512)99:0.1045040235,AF394529_Ordospora_colligata:0.3556069511)100:0.1865243865,(((((SRR16954902_Hamiltosporidium_magnivora:0.0012993579,SRR16954899_Hamiltosporidium_tvaerminnensis:0.0011732195)100:0.5406977425,((((((((SRR23214363_Nematocida_ausubeli:0.0482534697,SRR17622377_Nematocida_major:0.0493801792)76:0.0611071277,XR001214623_Nematocida_parisii:0.1849040428)100:0.4652495597,EF564602_Ovavesicula_popilliae:0.4426099381)100:0.3477337868,NG017174_Rozella_allomycis:0.6754674174)78:0.1068215572,(((((ERR3154977_Tubulinosema_ratisbonensis:0.0000022854,AF024658_Tubulinosema_acridophagus:0.0067459583)100:0.1750434162,(SRR489790_Anncaliia_algerae:0.1447156518,GU173849_Kneallhazia_carolinensae:0.0889520487)100:0.2731873561)100:0.0731958285,(MF278272_Fibrillaspora_daphniae:0.1766558011,AY364089_Fibrillanosema_crangonycis:0.0741322584)53:0.0285987202)100:0.2426575762,AY953292_Systenostrema_alba:0.5208555303)100:0.1984565831,(((((AY135024_Schroedera_plumatellae:0.0919222332,MN512229_Neoperezia_semenovaiae:0.0397933062)100:0.0556154110,AF484691_Bryonosema_plumatellae:0.0824629792)100:0.1716363648,((MN752317_Jirovecia_sinensis:0.1158401129,AJ581995_Bacillidium_vesiculoformis:0.0909695828)99:0.0327068725,AF484694_Pseudonosema_cristatellae:0.0802172379)85:0.0184956767)55:0.0155306582,AF484695_Trichonosema_pectinatellae:0.1914992208)89:0.0612273043,(JX463178_Pseudonosematidae_sp.:0.0386666509,(AY305324_Paranosema_locustae:0.0739440307,AF024655_Antonospora_scoticae:0.0674201252)100:0.3546640985)85:0.0951921410)84:0.0737498620)94:0.1198060800)85:0.0540200851,(((AJ252962_Flabelliforma_montana:0.0063553719,AY090069_Polydispyrenia_simuli:0.0000025293)100:0.0704156265,AF132544_Caudospora_palustris:0.0361128181)100:0.2562863668,KR704917_Myrmecomorba_nylanderiae:0.2908173305)97:0.0999217655)62:0.0652994279,((((((((((EU075347_Binucleata_daphniae:0.0154618938,KT950767_Agglomerata_cladocera:0.0017020810)100:0.0198998135,DQ641245_Senoma_globulifera:0.0246503852)100:0.1133531132,(MK053815_Pseudoberwaldia_daphniae:0.0190169136,AF439320_Gurleya_daphniae:0.1134753681)99:0.0166662804)100:0.0403159600,(AF394527_Larssonia_ob

tusa:0.0156522832,(MH645035_Conglomerata_obtusa:0.0014183543,AY090042_Berwaldia_schaefernai:0.0102258848)97:0.0022384546)100:0.0410537983)100:0.0820119073,((AY090067_Hazardia_milleri:0.0264256589,AY326268_Trichotuzetia_guttata:0.6340574444)100:0.0465786137,KX832080_Lanatospora_costata:0.1549169278)80:0.0294242096)81:0.0221325200,((AY090041_Marssoniella_elegans:0.0667540110,AY880951_Paraepiseptum_polycentropi:0.0694903241)86:0.0171228013,AY880953_Episeptum_circumscriptum:0.0408303421)100:0.0530717028)82:0.0240123901,(KT950766_Alfvenia_sibirica:0.1348622419,KC990122_Multilamina_teevani:0.2348299510)100:0.0914857820)93:0.0261180393,(FN794114_Octosporea_muscaedomesticae:0.0310447358,EF537880_Zelenkaia_sp.:0.1517445864)100:0.1957642542)100:0.1008378497,(((((JF826402_Amblyospora_bakcharia:0.0721882307,HM594269_Trichotosporea_pygopellita:0.0876401701)100:0.0708278561,(AY013359_Intrapredatorus_barri:0.1085863613,AY326269_Culicospora_magna:0.2060430602)99:0.0393414688)96:0.0240137948,AF027684_Edhazardia_aedis:0.1043646946)100:0.0685470076,(AF027683_Culicosporella_lunata:0.2422390995,AF483837_Hyalinocysta_chapmani:0.1871064858)100:0.2000473950)100:0.1524032155,EU664450_Andreanna_caspii:0.1312590309)100:0.1321396606)89:0.0370114608,((JF826419_Novothelohania_ovalae:0.2345871757,AY090065_Parathelohania_obesa:0.1025986450)98:0.0475389834,KF110990_Takaokaspora_nipponicus:0.2135945994)100:0.3169546912)100:0.3225655619)93:0.0732389839,SRR8476225_Astathelohania_contejeani:0.9716926742)80:0.0320719677)62:0.0273459869,GQ206147_Neoflabelliforma_aurantiae:0.4225018617)100:0.1147984091,(((((((SRR8495097_Cucumispora_dikerogammari:0.0958231988,KX364284_Hyperspora_aquatica:0.0454922737)100:0.1295947359,KU163282_Paradoxium_irvingi:0.0753711397)100:0.0346847578,DQ417114_Thelohania_butleri:0.0527039767)100:0.0623539816,HM140491_Myospora_metanephrops:0.0967847710)100:0.0836304788,((((((((SRR926312_Pseudoloma_neurophilia:0.0952068544,JQ062988_Ichthyosporidium_weissii:0.0677985901)100:0.0579158214,HM626203_Loma_salmonae:0.1465261306)100:0.0643191452,GQ203287_Glugea_hertwigi:0.0896394524)96:0.0168636949,(MT006314_Glugeidae_sp.:0.1680443775,MW077214_Fusasporis_stethaprioni:0.0516842256)77:0.0094881930)72:0.0097538182,AJ252958_Pleistophora_sp.:0.1164831586)100:0.0395965512,(((AF356223_Ovipleistophora_mirandellae:0.0346709337,KX099692_Pleistophora_beebei:0.0274897012)100:0.0265606136,(GU183263_Dasyatispora_levantinae:0.0571063667,KC137548_Heterosporis_sp.:0.0551893922)100:0.0231861705)100:0.0469799171,(AJ002605_Trachipleistophora_hominis:0.0394630502,XR552272_Vavraia_culicis:0.0333540041)100:0.0779474640)98:0.0169302239)100:0.0879966826,AY53

0532_Myosporidium_merluccius:0.1407055378)100:0.1002103729,((AJ438959_Dictyocoela_cavimanum:0.1027783275,MW377751_Unikaryon_panopei:0.1331711850)100:0.1306302717,JQ268567_Triwangia_caridinae:0.1943026206)71:0.0181541751)59:0.0204207869,((((AF356222_Kabatana_takedai:0.0676219601,MH911629_Inodosporus_octosporus:0.0538168112)99:0.0195582672,MF974572_Microsporidia_sp.:0.1065315768)100:0.0380554533,((AF364303_Tetramicra_brevifilum:0.0131319767,AY033054_Microgemma_caulleryi:0.0267823213)100:0.0564370255,GQ868443_Microsporidia_sp.:0.0426940700)100:0.0530918074)95:0.0255011920,(EU534408_Potaspora_morhaphis:0.0970586148,MG708238_Apotaspora_heleios:0.0619594883)100:0.1314275782)100:0.0926865154)86:0.0640042105)85:0.0544900999,((AY958070_Nadelspora_canceri:0.0096415889,MN935433_Ameson_herrnkindi:0.0295780956)100:0.7853415148,KX856426_Perezia_nelsoni:0.3602093328)88:0.1964533677)73:0.0503818248,(HM800849_Facilispora_margolisi:0.2670456534,MF429927_Microsporidium_sp.:0.2615014740)100:0.2668789765)100:0.3304866548)100:0.1799322487,((((((((SRR23214350_Pancytospora_philotis:0.0800866087,(KX424959_Pancytospora_epiphaga:0.0510661795,LC136798_Percutemincola_moriokae:0.0374253143)100:0.2168517810)100:0.1079356232,AJ302316_Orthosomella_operophterae:0.0837423593)90:0.0774093314,KX360142_Enteropsectra_longa:0.1782780486)97:0.0628781992,EU709818_Liebermannia_covasacrae:0.3511045746)100:0.2703574964,(((((((((SRR065293_Vittaforma_corneae:0.0716005919,(JX915758_Anostracospora_rigaudi:0.1273622596,L39109_Endoreticulatus_schubergi:0.1011501623)100:0.0876087293)52:0.0225323769,AF394525_Glugoides_intestinalis:0.2866656568)83:0.0308668002,GU130407_Crispospora_chironomi:0.0781307271)48:0.0357242504,AY233131_Cystosporogenes_legeri:0.0855683638)94:0.0382588863,FJ914315_Mrazekia_macrocyclopis:0.0901358378)95:0.0339202792,MN595900_Globosporidium_paramecii:0.0100668014)95:0.0594784048,(DQ675604_Euplotespora_binucleata:0.0832730518,GU130406_Helmichia_lacustris:0.3236589665)100:0.0730791450)100:0.3558603737,SRR926341_Agmasoma_penaei:0.5902622021)97:0.0416218281,GU126383_Anisofilariata_chironomi:0.3776814544)88:0.0624766202)94:0.0471809403,(JX915760_Enterocytospora_artemiae:0.0739895299,KT762153_Globulispora_mitoportans:0.0510152278)100:0.1587117348)65:0.0211585274,KX757849_Parahepatospora_carcini:0.1618536433)73:0.0469466533,((((FJ389667_Paranucleospora_theridion:0.1763596231,HG005137_Obruspora_papernae:0.1565482355)96:0.0287010097,U78176_Nucleospora_salmonis:0.0832004481)100:0.0901402672,(JX101917_Enterospora_nucleophila:0.1454874602,L07123_Enterocytozoon_bieneusi:0.2072854861)100:0.0568614394)100:0.0387261408,HE584635_Hepatospora_eriocheir:0.35

08670384)100:0.0772828820)100:0.2920163588)100:0.1773260440)99:0.1802956633,EU275200_Heterovesicula_cowani:0.7346172373)100:0.4783502727,MT510137_Nosema_bombycis:0.2859775967)100:0.1499259805,AF495379_Oligosporidium_occidentalis:0.0251747795)95:0.0192353697,DQ996241_Vairimorpha_necatrix:0.0359240190)100:0.0167039222,KR704648_Rugispora_istanbulensis:0.0412102351);

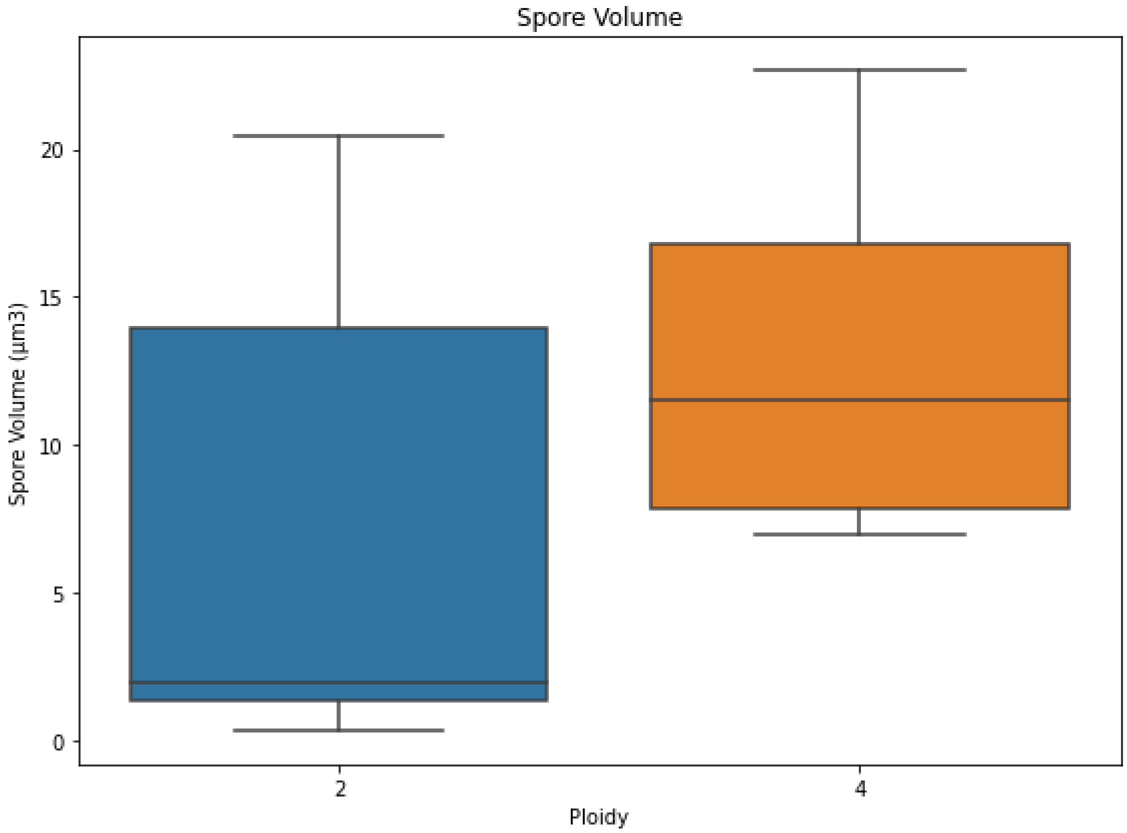

Figure S1: Box plot of spore volume in diploid (n = 10) and tetraploid species (n = 6), calculated using data from Bojko et al. (2022).

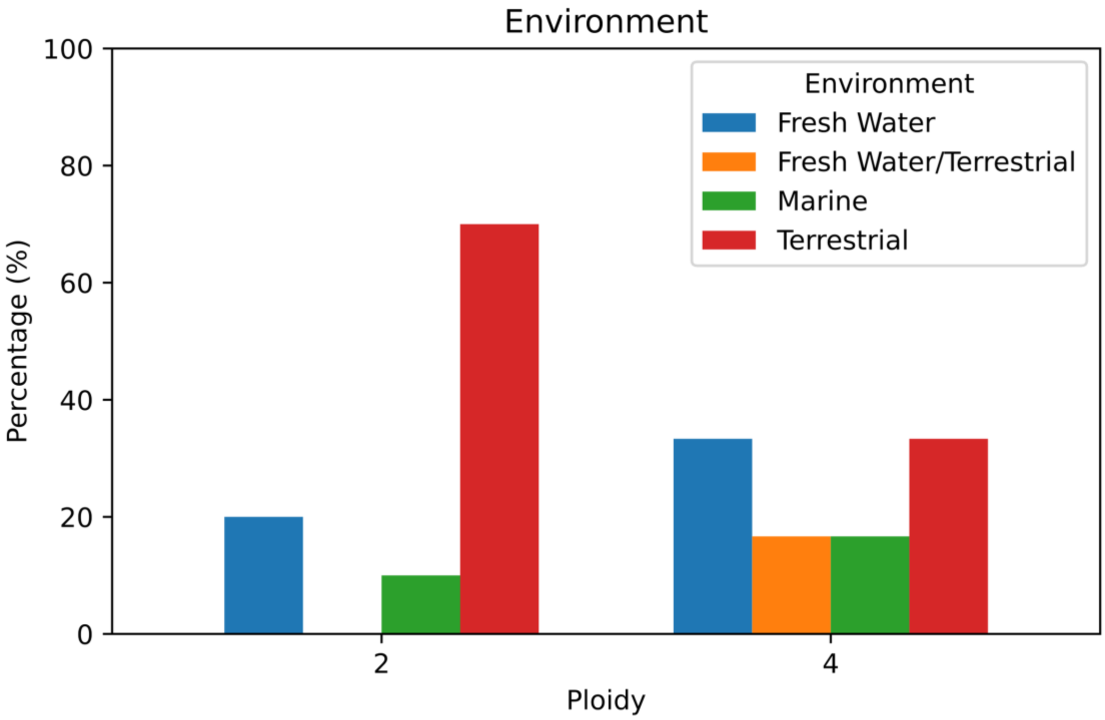

Figure S2: Bar plot of habitats the diploid (n = 10) and tetraploid species (n = 6) identified in this study are found in according to Bojko et al. (2022).

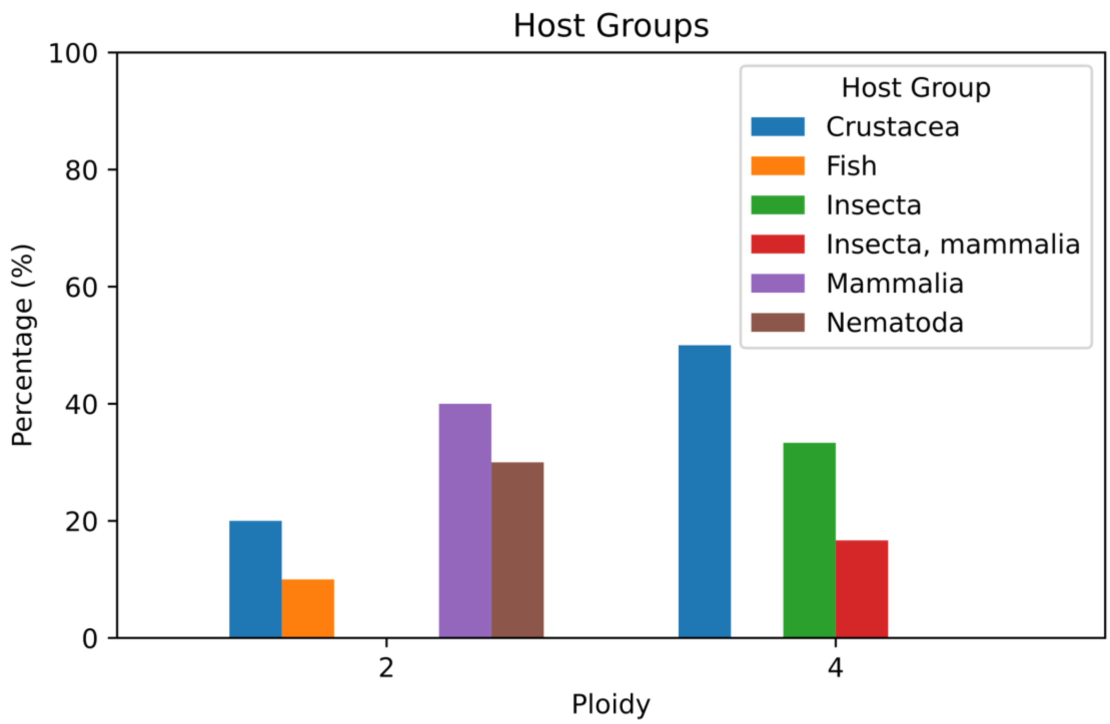

Figure S3: Bar plot of different host groups the diploid (n = 10) and tetraploid species (n = 6) identified in this study occur in, according to Bojko et al. (2022).

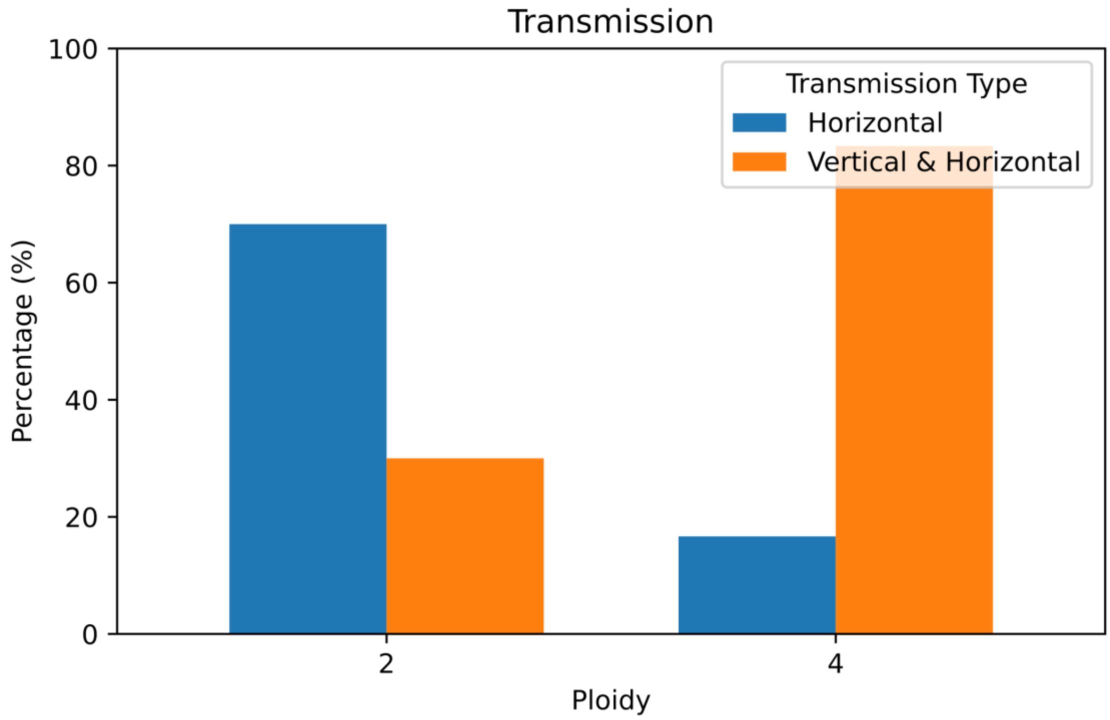

Figure S4: Bar plot of different transmission modes the diploid (n = 10) and tetraploid species (n = 6) identified in this study possess, according to Bojko et al. (2022).

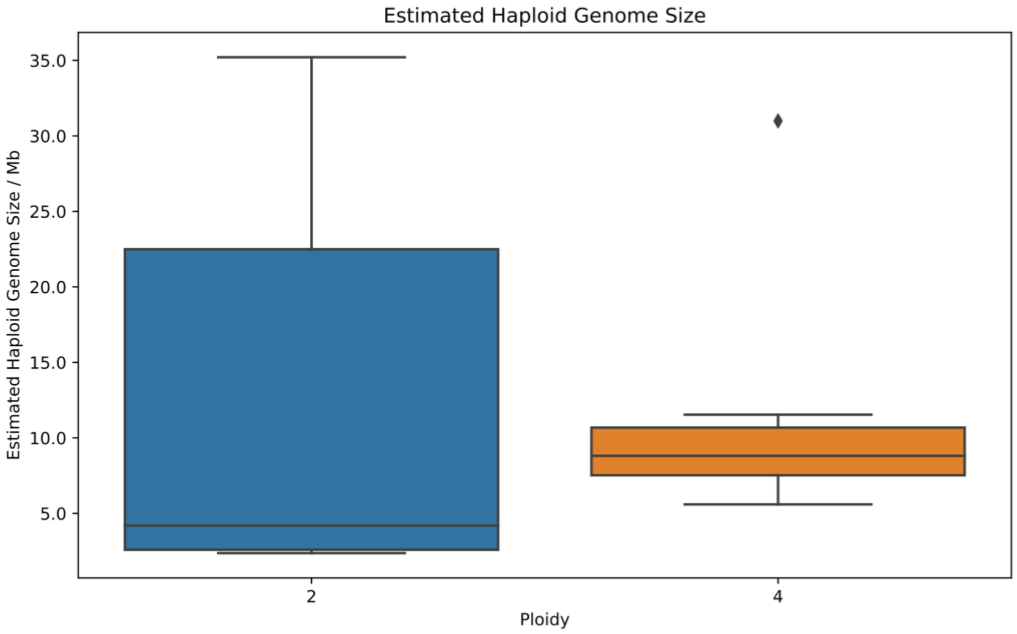

Figure S5: Box plot of average haploid genome size estimated for the diploid (n = 10) and tetraploid (n = 6) species identified in this study, using GenomeScope2.

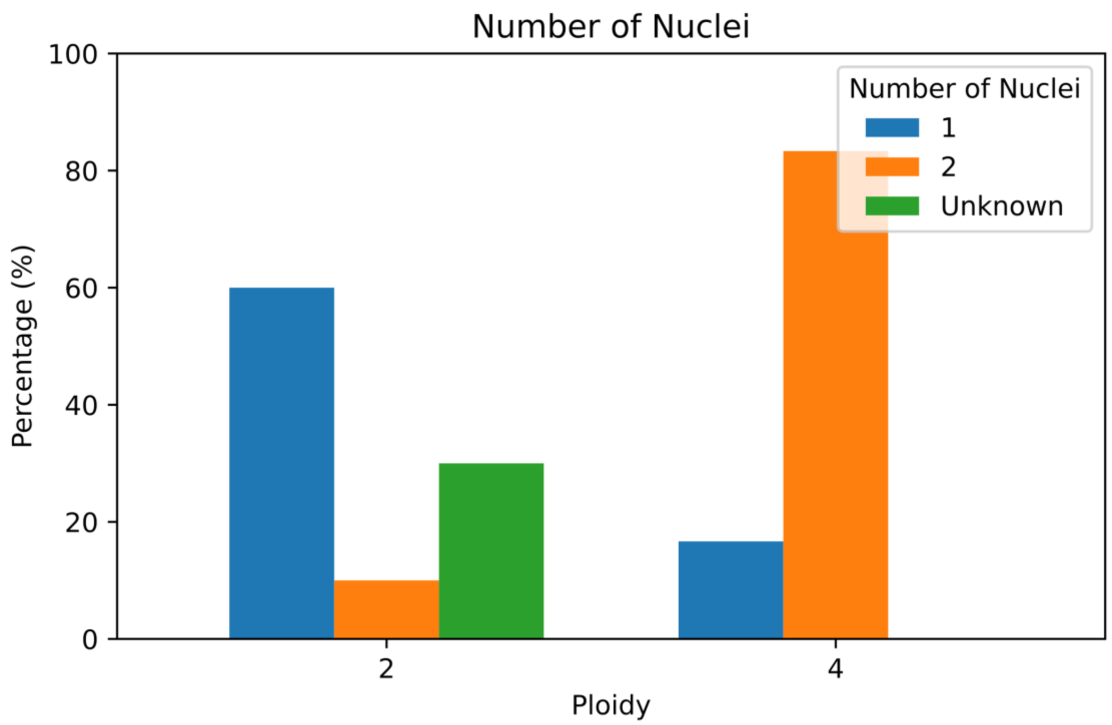

Figure S6: Bar plot of number of nuclei in the spores of the diploid (n = 10) and tetraploid species (n = 6) identified in this study, according to Bojko et al. (2022).
