# Supplementary material for: Polyploidy is widespread in Microsporidia
Source: Microbiol Spectr. 2024 Jan 12;12(2):e03669-23. doi: 10.1128/spectrum.03669-23 (PMC10845963; doi:10.1128/spectrum.03669-23)
Supplement: Table S2 — SRA accession numbers, species name, linear GenomeScope2 plots, transformed GenomeScope2 plots, and Smudgeplot plots of the 66 samples for which we were able to estimate ploidy in this study. [file spectrum.03669-23-s0002.pdf]

Table S2: SRA accession numbers, species name, linear GenomeScope2 plots, transformed GenomeScope2 plots, and Smudgeplot plots of the 66 samples for which we were able to estimate ploidy in this study.

Index	SRA Accession	Species	Estimated Ploidy	Genomscope2 Linear Plot	Genomscope2 Transformed Linear Plot	Smudgeplot	Comments
1	SRR17317295	Vairimorpha ceranae	4	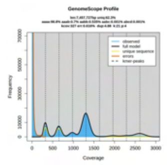	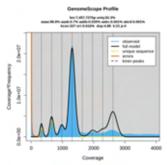	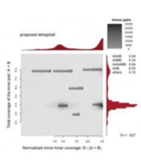	
2	SRR17317294	Vairimorpha ceranae	4	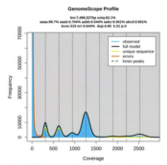	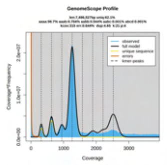	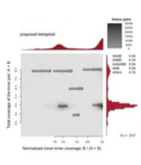	
3	SRR17317293	Vairimorpha ceranae	4	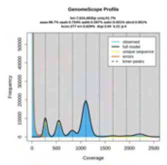	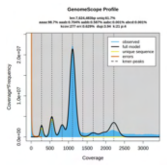	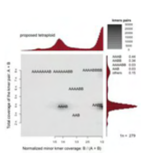	
4	SRR17317292	Vairimorpha ceranae	4	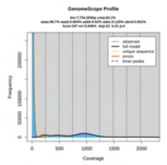	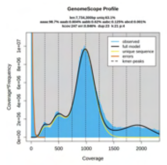	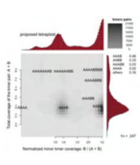	
5	SRR17317291	Vairimorpha ceranae	4	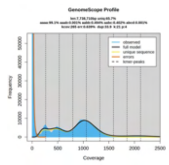	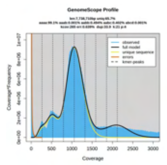	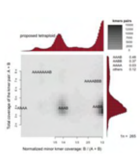	
6	SRR17317296	Vairimorpha ceranae	4	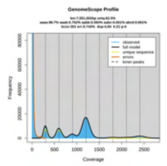	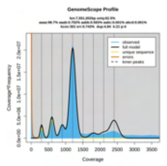	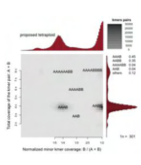	
7	SRR18590839	Vairimorpha ceranae	4	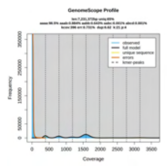	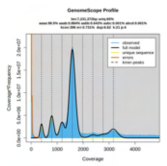	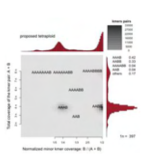	
8	SRR18590838	Vairimorpha ceranae	4	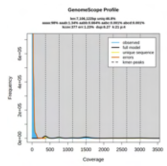	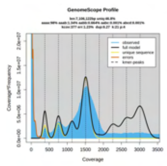	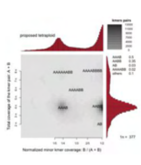	
9	SRR18590835	Vairimorpha ceranae	4	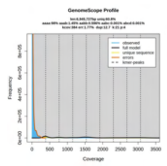	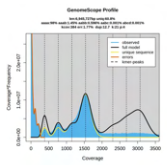	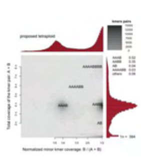	Low coverage contaminant

Index	SRA Accession	Species	Estimated Ploidy	Genomscope2 Linear Plot	Genomscope2 Transformed Linear Plot	Smudgeplot	Comments
10	SRR23560257	Encephalitozoon hellem	2	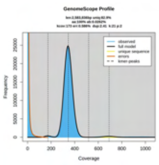	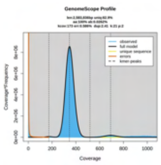	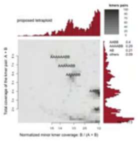	Genome is highly homozygous. The 4n smudge appears because imperfect duplications dominate over nearly negligible heterozygosity
11	SRR23560258	Encephalitozoon hellem	2	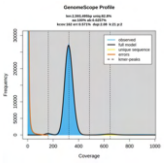	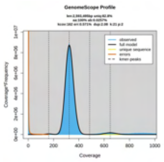	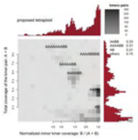	Genome is highly homozygous. The 4n smudge appears because imperfect duplications dominate over nearly negligible heterozygosity
12	SRR23560420	Encephalitozoon hellem	2	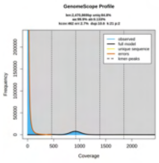	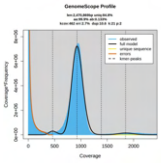	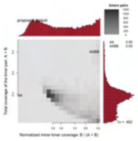	Genome is highly homozygous. Signal at the edges of the smudgeplot is noise
13	SRR24007516	Encephalitozoon intestinalis	2	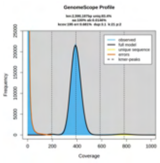	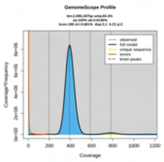	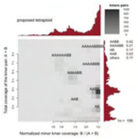	Genome is highly homozygous. The 4n smudge appears because imperfect duplications dominate over nearly negligible heterozygosity
14	SRR24007515	Encephalitozoon intestinalis	2	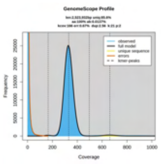	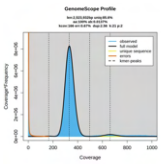	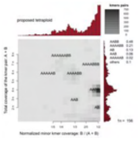	Genome is highly homozygous. The 4n smudge appears because

Index	SRA Accession	Species	Estimated Ploidy	Genomscope2 Linear Plot	Genomscope2 Transformed Linear Plot	Smudgeplot	Comments
							imperfect duplications dominate over nearly negligible heterozygosity
15	SRR24007514	Encephalitozoon intestinalis	2	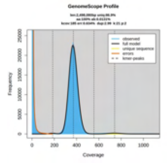	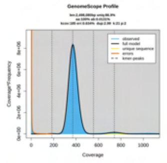	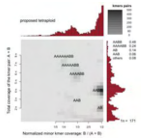	Genome is highly homozygous. The 4n smudge appears because imperfect duplications dominate over nearly negligible heterozygosity
16	SRR16954902	Hamiltosporidium magnivora	2	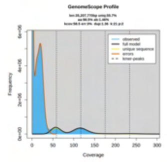	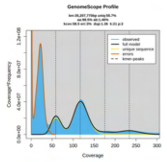	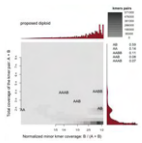	Low coverage contaminant
17	SRR16954901	Hamiltosporidium tvaerminnensis	2	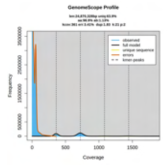	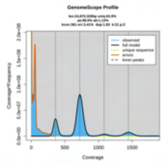	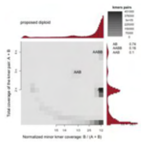	Low coverage contaminant
18	SRR16954899	Hamiltosporidium tvaerminnensis	2	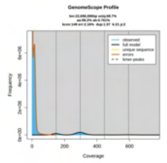	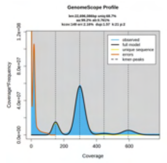	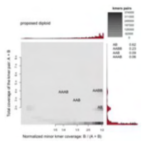	Low coverage contaminant
19	SRR16954898	Hamiltosporidium tvaerminnensis	2	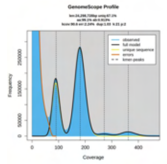	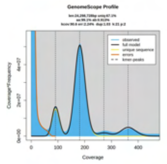	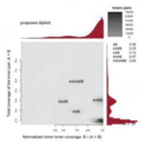	
20	SRR16954897	Hamiltosporidium tvaerminnensis	2	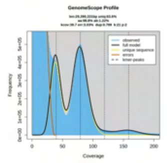	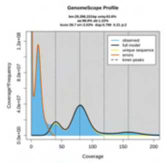	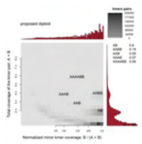	Low coverage contaminant
21	SRR16954895	Hamiltosporidium tvaerminnensis	2	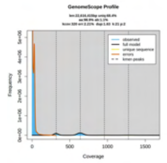	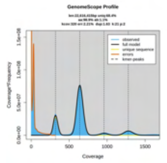	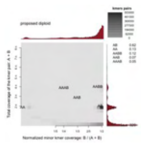	Low coverage contaminant

Index	SRA Accession	Species	Estimated Ploidy	Genomscope2 Linear Plot	Genomscope2 Transformed Linear Plot	Smudgeplot	Comments
22	SRR16954906	Hamiltosporidium tvaerminnensis	2	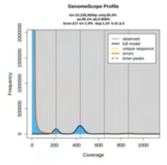	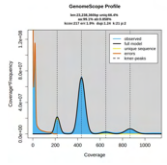	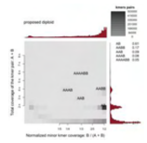	Low coverage contaminant
23	SRR16954905	Hamiltosporidium tvaerminnensis	2	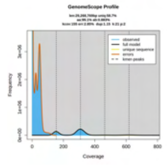	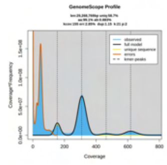	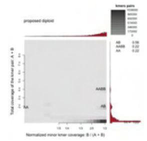	Low coverage contaminant
24	SRR16954904	Hamiltosporidium tvaerminnensis	2	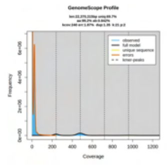	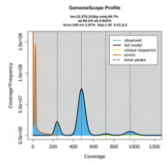	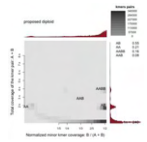	Low coverage contaminant
25	SRR23214363	Nematocida ausubeli	2	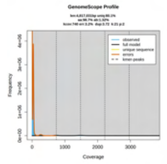	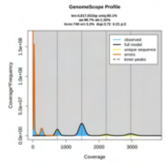	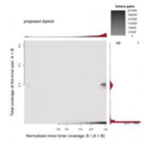	Low coverage contaminant
26	SRR23214355	Nematocida ausubeli	2	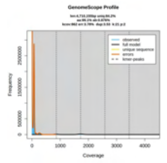	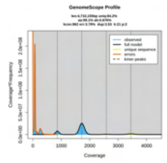	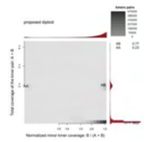	Sample has a low coverage contaminant
27	SRR23214353	Nematocida ausubeli	2	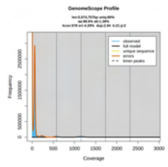	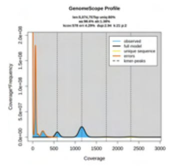	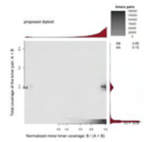	Low coverage contaminant
28	SRR23214358	Nematocida ausubeli	2	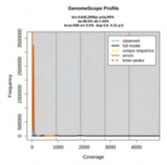	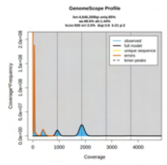	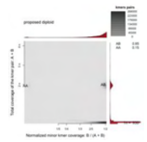	Low coverage contaminant
29	SRR17622377	Nematocida major	2	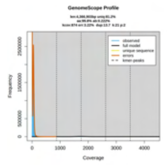	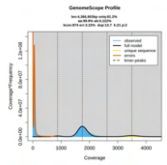	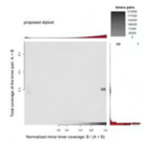	Low coverage contaminant
30	SRR23214350	Pancytospora philotis	2	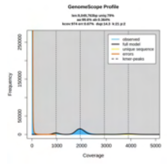	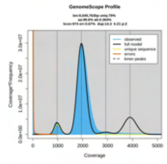	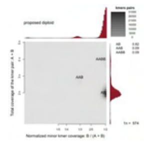	
31	ERR3154977	Tubulinosema ratisbonensis	4	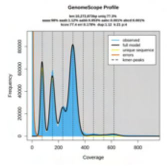	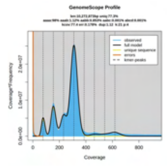	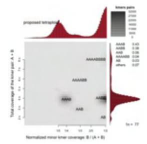	

Index	SRA Accession	Species	Estimated Ploidy	Genomscope2 Linear Plot	Genomscope2 Transformed Linear Plot	Smudgeplot	Comments
32	SRR2105612	Encephalitozoon cuniculi EcunIII-L	2	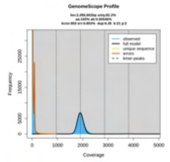	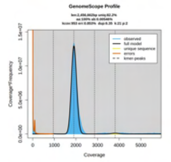	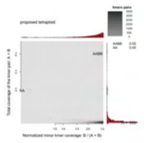	
33	SRR8476225	Astathelohania contejeani	4	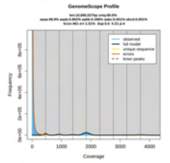	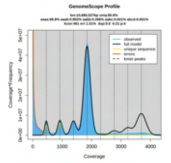	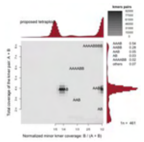	
34	SRR8476226	Astathelohania contejeani	4	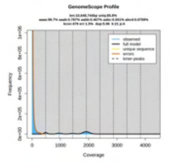	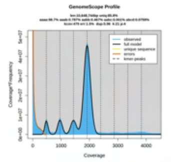	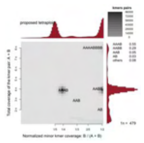	
35	SRR8495097	Cucumispora dikerogammari	4	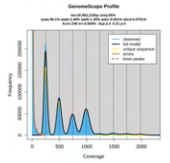	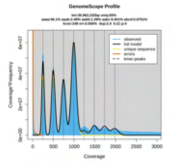	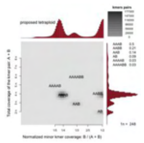	
36	SRR8495098	Cucumispora dikerogammari	4	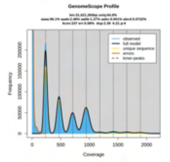	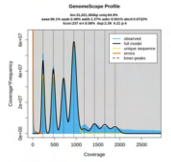	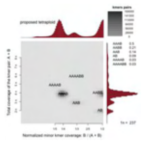	
37	SRR9597065	Hamiltosporidium tvaerminnensis	2	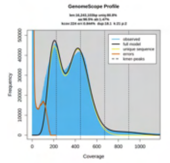	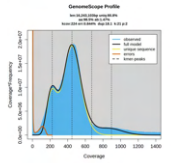	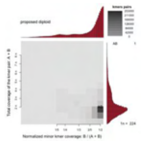	
38	SRR14017862	Encephalitozoon hellem	2	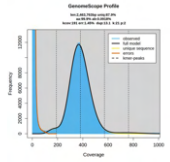	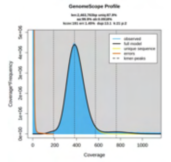	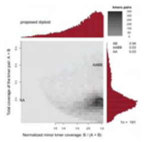	
39	SRR14062087	Encephalitozoon hellem	2	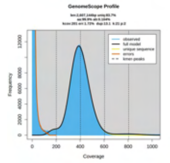	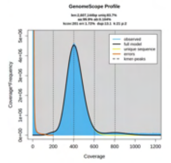	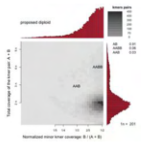	
40	SRR16954910	Hamiltosporidium tvaerminnensis	2	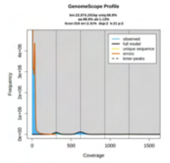	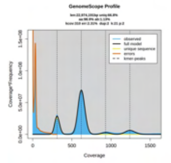	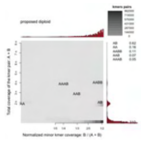	Low coverage contaminant
41	SRR16954909	Hamiltosporidium tvaerminnensis	2	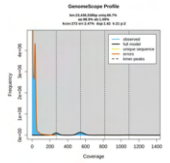	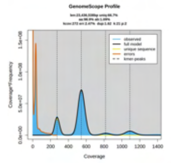	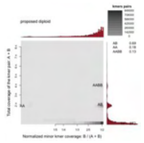	Low coverage contaminant

Index	SRA Accession	Species	Estimated Ploidy	Genomscope2 Linear Plot	Genomscope2 Transformed Linear Plot	Smudgeplot	Comments
42	SRR16954908	Hamiltosporidium tvaerminnensis	2	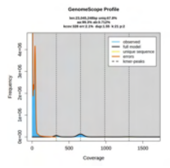	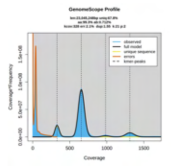	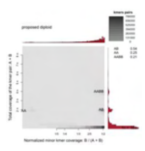	Low coverage contaminant
43	SRR543737	Anncaliia algerae	4	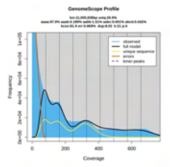	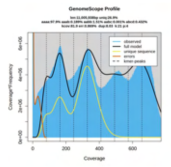	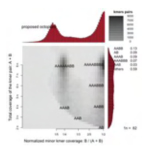	
44	SRR630040	Anncaliia algerae	4	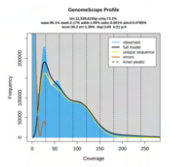	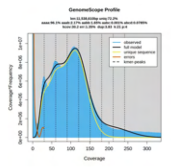	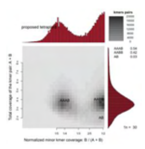	
45	SRR489790	Anncaliia algerae	4	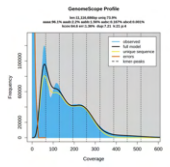	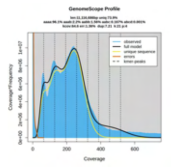	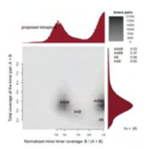	
46	SRR489792	Anncaliia algerae	4	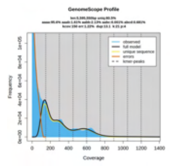	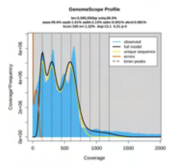	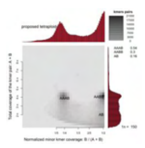	
47	SRR653671	Anncaliia algerae PRA109	4	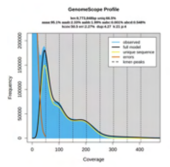	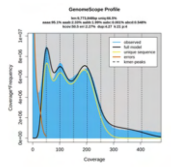	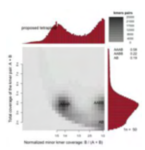	
48	SRR122310	Encephalitozoon cuniculi	2	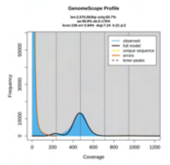	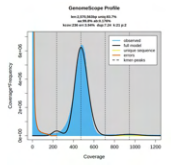	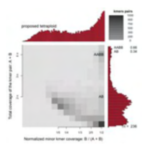	
49	SRR122316	Encephalitozoon cuniculi	2	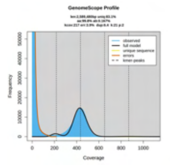	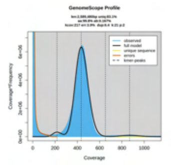	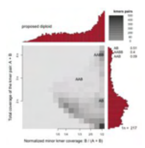	
50	SRR122317	Encephalitozoon cuniculi	2	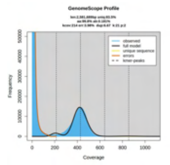	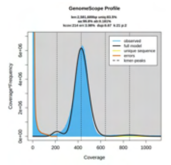	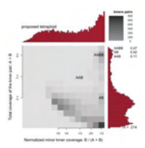	
51	SRR065293	Vittaforma corneae	2	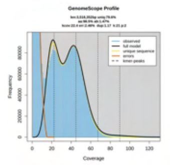	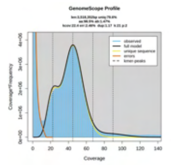	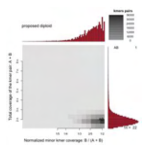	

Index	SRA Accession	Species	Estimated Ploidy	Genomscope2 Linear Plot	Genomscope2 Transformed Linear Plot	Smudgeplot	Comments
52	SRR926312	Pseudoloma neurophilia	2	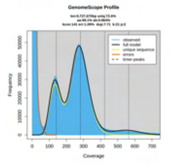	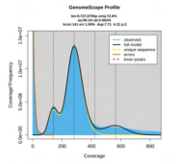	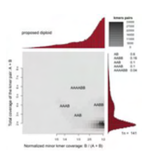	
53	SRR926320	Vairimorpha ceranae	4	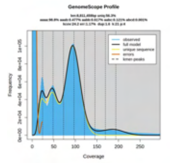	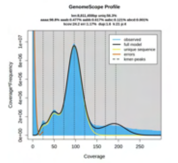	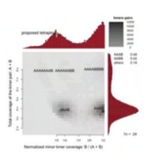	
54	SRR1596197	Vairimorpha ceranae	4	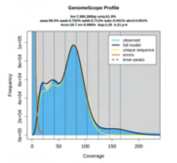	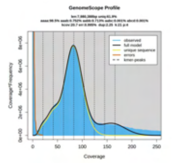	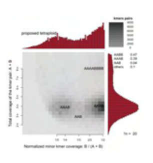	
55	SRR926341	Agmasoma penaei 2012 - Plaquemines Parish LA	4	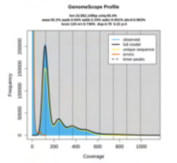	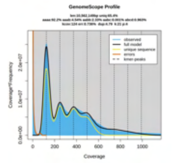	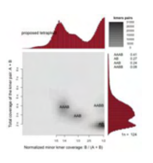	
56	SRR926400	Agmasoma penaei 2012 - Plaquemines Parish LA	4	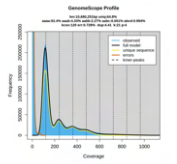	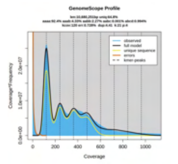	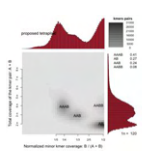	
57	SRR058692	Nematocida ausubeli	2	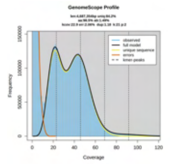	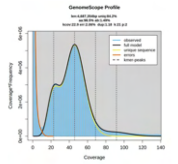	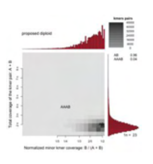	
58	SRR350188	Nematocida ausubeli	2	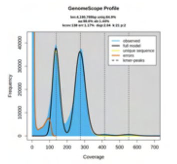	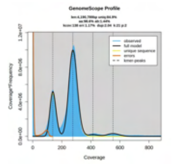	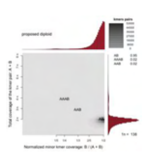	
59	SRR17853474	Encephalitozoon hellem	2	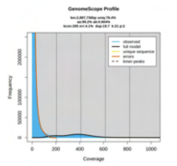	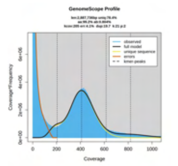	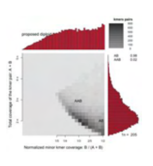	Smudgeplot is noisy, but genome appears to be highly homozygous
60	SRR17853475	Encephalitozoon hellem	2	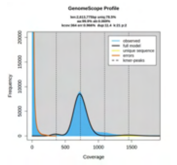	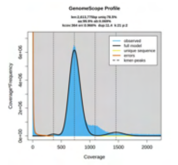	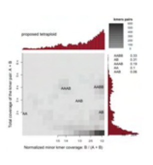	Smudgeplot is noisy, but genome appears to be highly homozygous

Index	SRA Accession	Species	Estimated Ploidy	Genomscope2 Linear Plot	Genomscope2 Transformed Linear Plot	Smudgeplot	Comments
61	SRR17865589	Encephalitozoon intestinalis	2	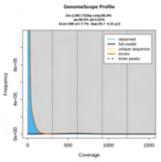	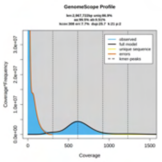	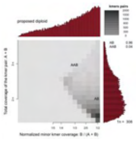	Smudgeplot is noisy, but genome appears to be highly homozygous
62	SRR17865590	Encephalitozoon intestinalis	2	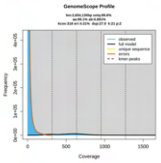	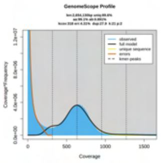	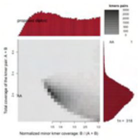	Smudgeplot is noisy, but genome appears to be highly homozygous
63	SRR17865591	Encephalitozoon intestinalis	2	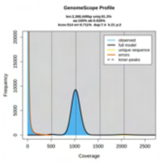	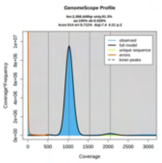	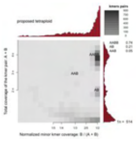	Smudgeplot is noisy, but genome appears to be highly homozygous. The reason for a smudge at 4n is that due to the high homozygosity, imperfect duplications appear overrepresented.
64	SRR17858634	Encephalitozoon cuniculi	2	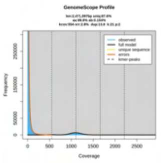	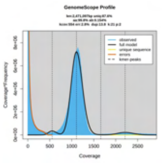	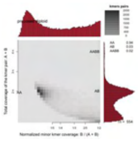	Smudgeplot is really noisy, but genome appears to be highly homozygous
65	SRR17858635	Encephalitozoon cuniculi	2	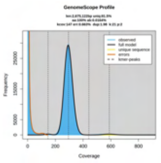	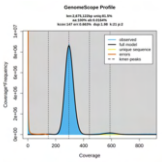	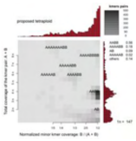	Smudgeplot is noisy, but genome appears to be highly homozygous. The reason for a smudge at 4n is that due to the high homozygosity, imperfect duplications appear overrepresented.

Index	SRA Accession	Species	Estimated Ploidy	Genomscope2 Linear Plot	Genomscope2 Transformed Linear Plot	Smudgeplot	Comments
66	SRR17858636	Encephalitozoon cuniculi	2	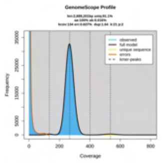	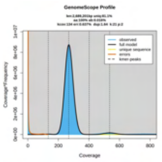	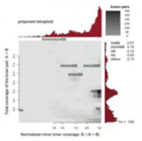	Smudgeplot is noisy, but genome appears to be highly homozygous. The reason for a smudge at 4n is that due to the high homozygosity, imperfect duplications appear overrepresented.
